# Giant reversible elongation upon cooling and contraction upon heating for a crosslinked *cis* poly(1,4-butadiene) system at temperatures below zero Celsius

**DOI:** 10.1038/s41598-018-32436-9

**Published:** 2018-09-24

**Authors:** Lu Lu, Jinbao Cao, Guoqiang Li

**Affiliations:** 1 Department of Mechanical & Industrial Engineering, Louisiana State University, Baton Rouge, LA 70803 USA; 2 Louisiana Multi-Functional-Materials Group, LLC, Baton Rouge, LA 70820 USA

## Abstract

Polymers with reversible elongation upon cooling (EUC) and contraction upon heating (CUH) enabled applications in actuators, fasteners, dampers, grippers, swimmers, sealants, etc. With the current working temperature being limited to mainly above zero Celsius, applications for subzero Celsius environments are obstructed. In addition, current reversible actuation needs a constant tensile load, or for the best case, under zero tensile load. Reversible EUC and CUH under compressive load is almost impossible and has not been explored. In this work, a *cis* poly(1,4-butadiene) based system has been developed. Actuated below zero Celsius, 69% EUC occurred under a tensile load; and 6.2% EUC and 17.9% CUH occurred under 0.05 MPa compressive load. The reversible actuation was driven by both entropy and enthalpy, which was validated by a series of characterization tools.

## Introduction

Shape memory polymers (SMPs) are smart materials, which can be deformed into temporary shapes and restore their permanent shapes by external stimuli on demand^1–3^. Two-way shape memory polymer (2W-SMP) represents a special class of SMPs. Features make 2W-SMP so unique and attractive include: (1) 2W-SMP behaves thermally opposite to common physics, i.e. expands upon cooling and contracts upon heating; (2) the expansion and contraction in response to temperature change can undergo reversible cycles in a continuous mode once triggered. Two-way shape memory effect (2W-SME) was initially reported in 2001 for liquid crystalline elastomers^4^. In 2008, Mather *et al*. observed the 2W-SME in a semicrystalline polymer network – crosslinked poly(cyclooctene)^5^. Later on, other polymer systems were also demonstrated to display 2W-SMEs with varying actuation abilities and working temperature ranges, which includes poly(Ɛ-caprolactone)^6–13^, oligo(pentadecalactone)^14,15^, ionomer Nafion^16^, poly(octylene adipate)^17–19^, poly(ethylene-*co-*vinyl acetate)^20,21^, polyurethane^22–25^, and more^5,26–32^.

Owing to the intriguing properties of 2W-SMPs, soft actuators, morphing structures, fastening devices, optical gratings, grippers, fixators, swimmers, and artificial muscles^10,17,25,33,34^ can be designed using 2W-SMPs to meet different application requirements. However, there is one major limitation for the previously developed 2W-SMPs. The working temperature is mainly restricted to above 0 °C and the lowest is −20 °C with reversible strain actuation only 30% under tensile load^13,24^. This is far from sufficient to realize the applications of 2W-SMPs in areas that require large reversible actuation below 0 °C. Therefore, the first objective of this study is to develop a polymeric system with giant 2W-SME at temperatures below 0 °C.

External tensile load is generally required for the 2W-SME. However, certain reports indicate that the tensile load can be replaced by constructing multiphase networks^21,25^ or introducing internal tensile stress to the stable network through tensile programming^7,35^. Polymeric materials with reversible elongation upon cooling (EUC) and contraction upon heating (CUH) under compressive load have not been achieved, although their fundamental science, properties and applications are far more attractive. Therefore, it is the second objective of this work to develop a polymeric system with reversible EUC and CUH under compressive load.

In addition to the well-known applications of 2W-SMPs, 2W-SMPs with EUC and CUH may find additional applications, particularly with actuation at subzero Celsius temperatures. For example, low temperature cracking is unavoidable in asphalt pavement^36^, and both cracking and joint are inevitable in Portland cement concrete pavement^37^. These joints or cracks, if not sealed properly, will entrap debris and allow water to penetrate into the surface layer and layers beneath, leading to premature structural failure. Clearly, the sealant material needs to perform thermally opposite to construction materials, i.e., EUC and CUH, to keep tight sealing in the winter and to avoid sealant squeezing out of the joint/crack space in the summer. Due to the reversible EUC and CUH, the 2W-SMPs may be suitable for application as sealant to seal joints and cracks in pavements and bridge decks. The EUC is particularly useful because low temperature cracking is a quite challenge problem and has not been adequately solved up to the present time. Similar applications may be found as gasket for pipelines, which is a critical component for oil and gas transport where the pipeline is exposed to daily temperature fluctuations.

In this study, we synthesized a crosslinked poly(1,4-butadiene) (97% *cis* content) with 3 wt% dicumyl peroxide. The reversible EUC and CUH under a constant tensile load was investigated, particularly actuation below 0 °C. The reversible actuation without the external tensile load, and even under an external compressive load, was then studied. The mechanisms driving the giant reversible actuation were investigated through a series of physical, mechanical, and chemical characterizations.

## Results and Discussions

### Reversible EUC and CUH below 0 °C

**Figure 1:**
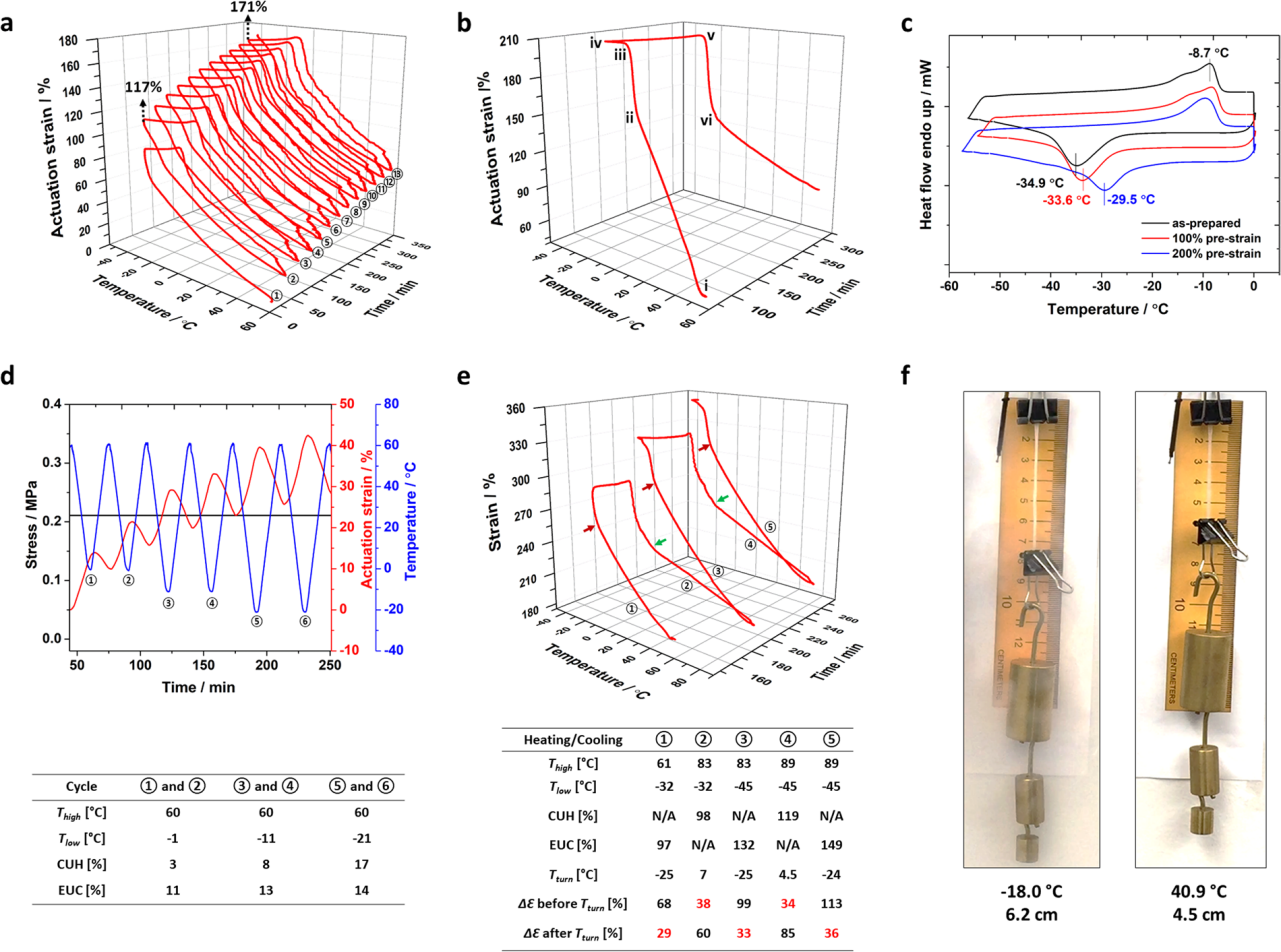
The reversible EUC and CUH of the cPBD under tensile loads. (**a**) Multiple heating and cooling cycles acquired with dynamic mechanical analyzer. The pre-strain was 125%. A total of 13 cycles were conducted. (**b**) A study with slower heating and cooling rate (1 °C min^−1^ for **b** vs. 10 °C min^−1^ for **a**). The pre-strain for the specimen was 167%. The turning points above and below the crystallization zone were clearly marked. (**c**) Differential scanning calorimeter scans of three specimens with different pre-strains. (**d**) Actuations above crystallization zone under 0.21 MPa tensile load. For each temperature window, two cycles were conducted, i.e., three temperature windows and six cycles. (**e**) Actuations within different temperature windows under 0.19 MPa tensile load. A total of five temperature windows were used. (**f**) Visualization of the actuation of the cPBD.

Several examples of the EUC and CUH of the crosslinked poly(1,4-butadiene) (cPBD) below 0 °C are shown in Fig. 1 (more examples are given in Figs S4–S6, Supplementary Information). For the tests, pre-stretched cPBD specimens were subjected to stress and temperature changes with time. The strain change with time was recorded by dynamic mechanical analyzer (DMA).

In Fig. 1a, the heating/cooling rate was 10 °C min^−1^. The specimen was first tensile stretched to 125% strain. Actuation of 97% EUC and 78% CUH were achieved under 0.21 MPa tensile stress when sweeping temperature between −35 and 60 °C. The temperature window was then expanded to −45 to 65 °C and the test results indicated a 106% EUC and 101% CUH. The results at such low temperature (−45 °C) pushed the working temperature limit of cPBD to far below 0 °C while also achieving significant actuation (>100%). Additionally, immersing the specimen in rain water at room temperature for 4 months or boiling it in saturated salt water for 2 hours has NO influence on the performance, which proves the possibility of applying cPBD in wet and snowy outdoor environment such as sealant in pavement.

In Fig. 1a, the actuation strain increased from 117% to 171% after ten thermomechanical cycles. This indicates a creep effect of (171 − 117%)/10 = 5.4% on average for one cycle. For most 2W-SMP systems, creep effect, or hysteresis actuation, is a disadvantage. However, when applying cPBD as joint sealant, the hysteresis actuation is needed. Concrete pavement joints double their widths after 5–10 years of service. The hysteresis actuation of cPBD will accommodate the gradual joint widening. Furthermore, the creep effect directly relates to the external load during the 2W-SME study. It is generally true that the higher the external tensile load, the larger the creep effect. This provides a strategy to tune the creep according to the target application. It is worth mentioning that the creep effect will not lead to complete permanent strain. After load removal at the end of the test, more than 95% strain increase from creep will be recovered, i.e., the majority of the creep is actually viscoelastic deformation.

To compare the influence of heating/cooling rate on the 2W-SME and visualize the details of strain changes during temperature sweeping, 1 °C min^−1^ heating/cooling rate was used in Fig. 1b. From point i (60 °C) to ii (−10 °C), the actuation strain increased from 48% to 154% within 66.5 mins (1.6% min^−1^). From point ii (−10 °C) to iii (−23 °C), the strain increased from 154% to 205% within 11.6 mins (4.4% min^−1^). The slope change during cooling indicates two possible contributing mechanisms. From point iii (−23 °C) to iv (−50 °C), the specimen contracted 3.7% due to the positive coefficient of thermal expansion (CTE). The specimen should have been fully crystallized at −23 °C under such big strain (167% pre-strain + 205% current reading = 372%). It indicates that large pre-strain (e.g. 372%) is not preferred for applications requiring reversible actuation within −23 °C to −50 °C. From point iv (−50 °C) to v (0 °C), the specimen elongated 7.5% due to the positive CTE. With increase from 0 °C to 6 °C (point vi), the specimen experienced a quick shrinkage of 64%. Further increasing the temperature to 60 °C led to a relatively slower strain decrease of another 58%. Therefore, with 1 °C min^−1^ heating/cooling rate, overall 153% EUC and 114% CUH were achieved with obvious slope changes of the strain curves. As compared with 10 °C min^−1^ heating/cooling rate, the reversible actuation with 1 °C min^−1^ heating/cooling rate is larger. It is due to the larger pre-strain (125% vs. 167%) and the creep effect (Fig. S10, Supplementary Information).

The melting and crystallization temperatures of the cPBD are −8.7 and −34.9 °C, respectively (Fig. 1c). However, in Fig. 1a,b, tremendous actuation can be seen when sweeping temperature above 0 °C and there are clear slope changes in the strain curves around the crystallization temperatures for cooling curves and around the melting temperatures for the heating ones. To better understand the mechanism, different working temperature windows were investigated (Fig. 1d,e) with heating/cooling rate at 5 °C min^−1^. The highest temperature (*T*_*high*_), the lowest temperature (*T*_*low*_), CUH, EUC, the temperature at the slope changing point (*T*_*turn*_) and the strain change (*ΔƐ*) before and after *T*_*turn*_ for each cycle are listed in the in-set tables below each figure. In Fig. 1d, the actuation of cPBD was studied at temperatures above its crystallization transition. The external tensile stress was 0.21 MPa. The CUH was 3–17% and EUC was 11–14% at relatively low pre-strain ~69%. It shows that cPBD has clear 2W-SME above its crystallization temperature, which was not due to crystallization/melting transition (the well acknowledged mechanism for semicrystalline shape memory polymers). Since cPBD was in amorphous state within these temperature windows, it is believed that entropic elasticity is responsible for the reversible actuation above the crystallization/melting transition.

The temperature window was expanded in Fig. 1e and the external tensile stress was 0.19 MPa. The slope changing points are labeled with either red arrows for heating cycles or green arrows for cooling ones. For the cooling cycles, the temperatures at the turning points (*T*_*turn*_) were from −24 to −25 °C, around the crystallization temperature of cPBD. Below *T*_*turn*_ in the cooling cycles, the slopes of the strain curves increased, indicating enhanced contribution of crystallization induced elongation. For the heating cycles, the specimen first expanded slightly before reaching to its melting point, due to the positive CTE. Then contraction upon further heating happened with a steeper slope due to the significant contribution of melting induced contraction. After reaching 4.5 to 7 °C, the slopes of the strain curves decreased. The continuous contractions after *T*_*turn*_ were due to entropic elasticity. The melting/crystallization contributions to the strain actuations in the in-set table are labeled in red. It can be seen that the melting/crystallization contribution was limited as compared to entropy elasticity.

The giant EUC and CUH were demonstrated (Movie S1, Supplementary Information). The setup and dynamic process are shown in Fig. S7 and Table S2 in the Supplementary Information. Two screenshots are shown in Fig. 1f. The specimen with a mass of 0.067 g was tested with 130 g (equals 0.18 MPa) weight hanged, indicating the cPBD can easily lift up and actuate under the weight which is 1,940 times heavier than itself. The original length of the specimen was 1.0 cm and it became 4.2 cm after loading 130 g weight. The length became 6.2 cm during cooling and 4.5 cm during heating (100% × (6.2 − 4.5)/1 = 170% strain actuation).

### Reversible EUC and CUH under Compressive Load

**Figure 2:**
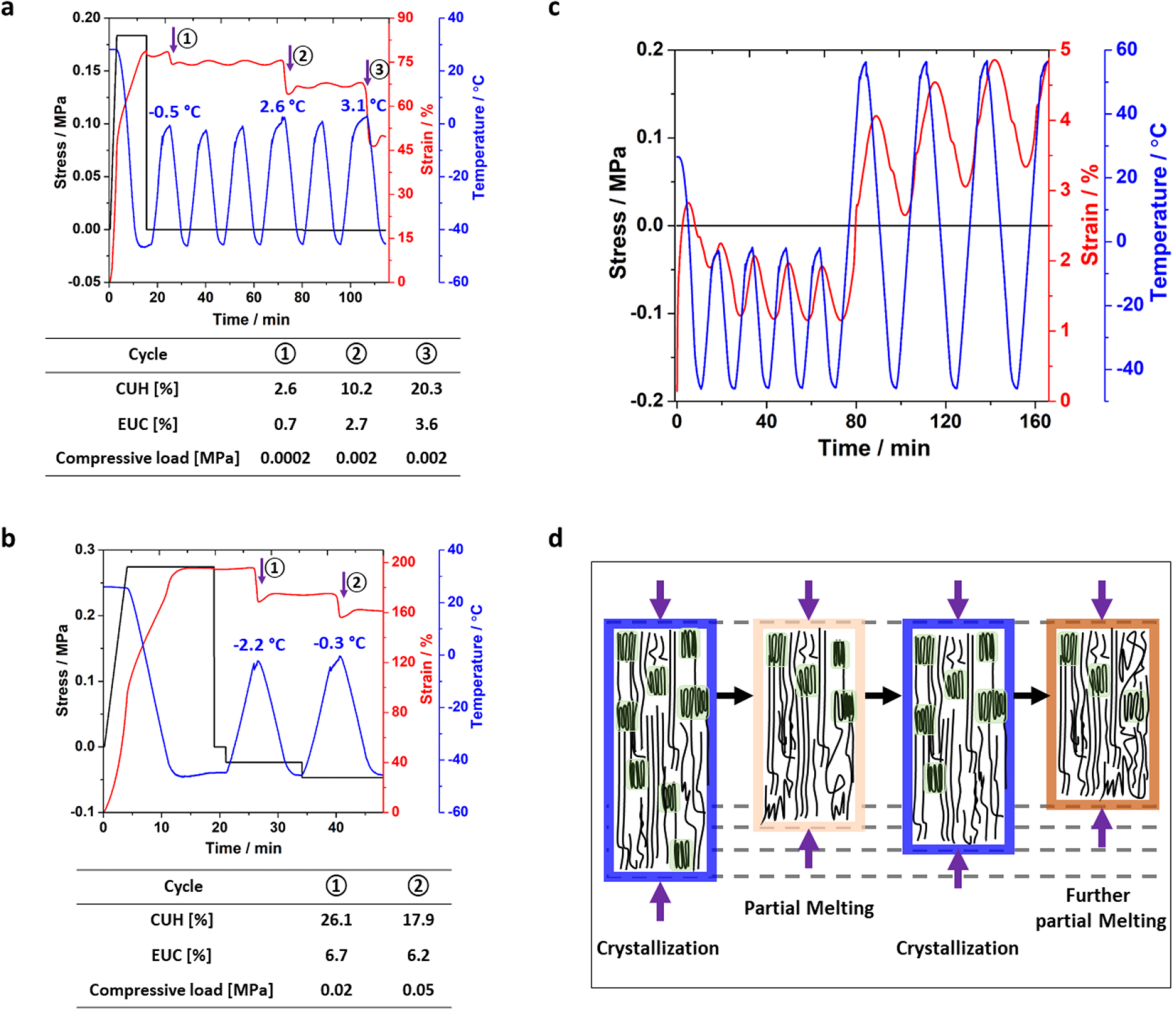
The reversible EUC and CUH of the cPBD under compression. (**a**) Low compressive load of 0.0002 MPa and relatively low programming level of 76.9%. (**b**) Gradually ramping up the compressive load to 0.02 MPa and 0.05 MPa with relatively larger programming level of 194.6%. (**c**) The coefficient of thermal expansion (CTE) study. (**d**) Illustration of the EUC and CUH under compressive load of the cPBD.

While reversible EUC and CUH under tensile load and without any load have been explored by researchers, polymers with reversible EUC and CUH under compressive load has never been investigated. Such actuations under compressive load are needed in many applications. For example in biomimetic crack healing, compressive load helps crack closure and thus ultimate healing^33^. In Fig. 2a, the specimen was first stretched to 76.9% strain. Then, a compressive stress of 0.0002 MPa was applied, and the specimen was subjected to thermomechanical cycles. Three cycles with EUC and CUH can be seen. The actuation values are summarized in the in-set table. By keeping the low temperature the same (−45 °C) and elevating the high temperature from −0.5, to 2.6, and to 3.1 °C, the CUH and EUC values both increased. In the second test (Fig. 2b), the compressive load was increased from 0.02 to 0.05 MPa. The reversible CUH and EUC were still achieved. It is confirmed that the CUH was due to reversible actuations, not specimen buckling and compressive strain by the compressive load. The applied compressive load was 0.5 N, while the buckling load was 35 N. The compressive strain by the compressive load was 0.073%, which is much smaller than the CUH (detailed calculations in the Supplementary Information). In Fig. 2c, the cPBD shows positive CTE behavior, which expands upon heating and contracts upon cooling (detailed discussion in Supplementary Information). It indicates that the CUH and EUC do not come from the negative CTE and programming is the requirement for the specimen to display such reversible actuation. In Fig. 2d, the mechanism for actuation under compressive load is proposed. Partial melting induced the CUH effect and cooling below the crystallization temperature led to the elongation of the specimen with only partial strain recovery compared to the state before heating. Further heating and additional partial melting led to contraction upon heating again. The energy stored within crystals and bond length changes during cooling served as the driving force for the remarkable reversible actuation subjected to compressive load.

### Mechanisms for the Reversible EUC and CUH of the cPBD

**Figure 3:**
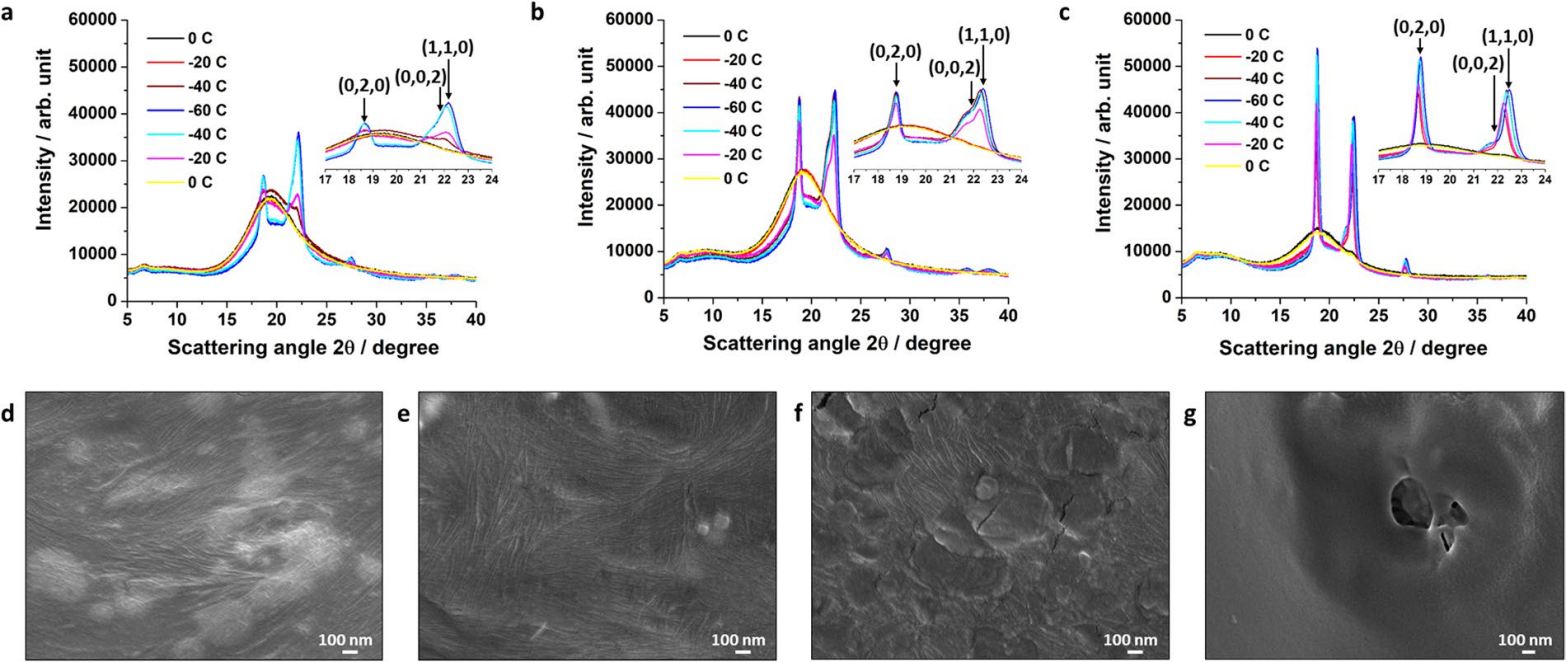
Crystallization-melting transitions of the cPBD elucidated with *in situ* X-ray diffraction (XRD) and cryogenic scanning electron microscopy (cryo-SME). (**a**–**c**) XRD studies of three specimens with increasing pre-strains and the corresponding zoom-in views with the Miller indices. The Y scales were set to be in the same range for ease of comparison. (**a**) As-prepared specimen. (**b**) Specimen with 100% pre-strain. (**c**) Specimen with 200% pre-strain. **d**–**g**), Cryo-SEM observations of the as-prepared specimen at different temperatures. (**d**) −60 °C. (**e**) −40 °C. (**f**) −20 °C. (**g**) 0 °C.

The crystallization-melting transition of the cPBD was characterized with *in situ* X-ray diffraction (XRD) and cryogenic scanning electron microscopy (cryo-SEM) (Fig. 3). As-prepared, 100% and 200% tensile stretched specimens were subjected to XRD scans. All specimens experienced a cooling cycle from 0 to −20, −40 and −60 °C, followed by a heating one from −60, to −40, −20, and 0 °C so that the melting and crystallization transitions can be captured. For the as-prepared specimen (Fig. 3a), a small lump at 21.9° appeared at −40 °C, indicating the presence of crystalline phase. At −60 °C, two sharp peaks at 18.7° and 22.2° with a small peak at 27.7° indicate more crystal formations. During heating, peak intensity drop can be observed by heating the specimen to −20 °C. This crystal melting correlates well with the cPBD melting transition from −22 to −5 °C based on DSC (Fig. 1c). At 0 °C, the specimen returned to its amorphous state.

For the 100% stretched specimen (Fig. 3b), three sharp peaks appeared at the same positions as compared to the as-prepared specimen at −40 °C with stronger diffraction intensities. Cooling to −60 °C then heating to −40 °C did not have any influence on the diffraction pattern, suggesting complete crystallization at −40 °C. Partial crystal melting at −20 °C led to the diffraction intensity drop and the specimen returned to amorphous state at 0 °C.

Similarly, the diffraction peaks of the 200% stretched specimen become sharper and stronger, indicating the increased crystallinity with the increase in the pre-strain (Fig. 3c). The crystal formation started above −20 °C. Further cooling to −40 and −60 °C and heating back to −40 °C showed no influence on the crystallinity since the specimen was fully crystalized at −40 °C. Slight melting at −20 °C and amorphous state at 0 °C were then observed.

Clearly, increase in the pre-strain will elevate the crystallization/melting temperatures (also noticed in Fig. 1c) as well as the crystallinity, and consequently enhance the reversible EUC and CUH (Fig. 1). In other words, the actuation of the cPBD is tunable depending on the requirement of the working temperature range and actuation level for a specific application.

The crystallinities at two selected temperatures were determined by comparing the peak area between crystalline and amorphous phases using PDXL software (Table 1). Pseudo-Voigt peak function was used for peak fitting. At each temperature, the crystallinity increased with the increase in pre-strain. For each specimen, lowering temperature from −20 to −40 °C leads to crystallinity increase. The peak at 21.9° was generated from (0, 0, 2) plane, therefore the d-spacing for the 21.9° peak and the corresponding strain change (ΔƐ) upon stretching were calculated. In consideration of the modulus of the specimen at −40 °C (165 MPa from Fig. S1, Supplementary Information), the residual stress within the crystals can be calculated and is given in Table 1. The residual stress generated and stored upon stretching and cooling below the crystallization transition is believed to be the driving force for the reversible actuation with zero load or with compressive load.

**Table 1:** Analysis of the XRD results.

	Crystallinity at −20 °C (%)	Crystallinity at −40 °C (%)	d-spacing for 21.9° peak at −40 °C (Å)	ΔƐ	Macroscopic residual stress (MPa)
As-prepared	6.78	13.13	4.0496		
100% stretched	11.53	37.48	4.1015	0.01286	2.11
200% stretched	47.47	68.03	4.1060	0.01396	2.30

The surface morphology of the as-prepared specimen was visualized with cryo-SEM at −60, −40, −20, and 0 °C. At −60 °C (Fig. 3d) and −40 °C (Fig. 3e), nanoscale fibrous structures can be seen throughout the entire surface. When increasing temperature to −20 °C, the amount of the fibrous nanostructures reduced significantly and multiple plateau areas appeared, indicating melting of crystalline domains (Fig. 3f). At 0 °C, fibrous structures completely disappeared which means that the specimen was at its molten state (Fig. 3g). This result correlates well with the observations from XRD and DSC studies.

**Figure 4:**
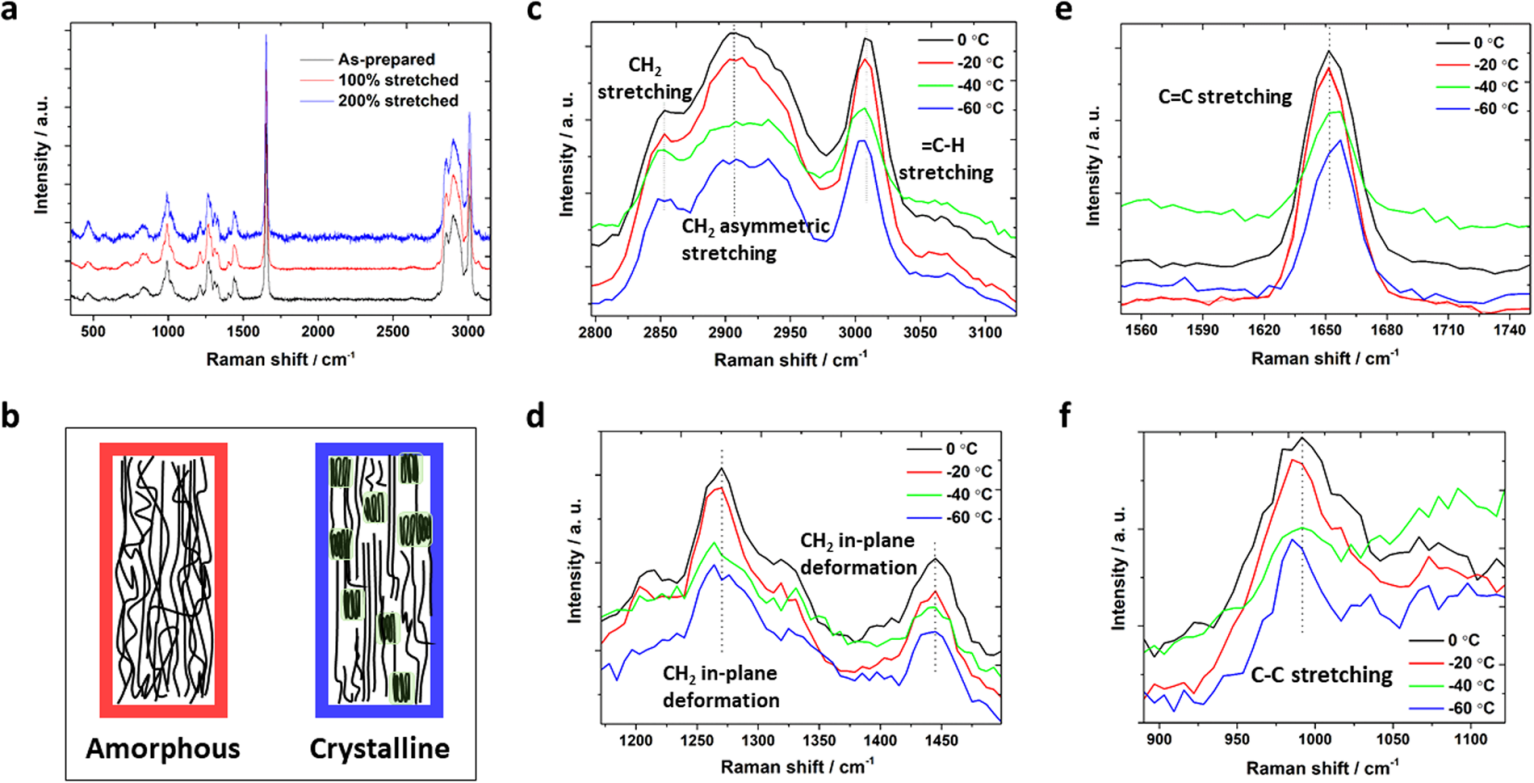
Chemical bond length change of cPBD characterized with Raman spectroscopy. (**a**) The Raman spectrum of three specimens with different pre-strains scanned at room temperature. (**b**) Illustration of the microscopic structure change of cPBD during crystallization. (**c**–**f**) Zoom-in views of four Raman shifting regions acquired with *in situ* Raman Spectroscopy during cooling from 0 to −20, −40 and −60 °C for the as-prepared specimen. (**c**) CH_2_ and =C-H stretching region. (**d**) CH_2_ in-plane deformation region. (**e**) C=C stretching region. (**f**) C-C stretching region.

Raman spectroscopy was conducted to monitor whether there was any peak shifting or bond length change during tensile stretching or crystallization (Fig. 4). No peak shifting was noticed between the specimens with different pre-strains at room temperature (Fig. 4a). Upon crystallization, it is believed that bond length change will be involved to store energy (Fig. 4b). When dropping temperature of the as-prepared specimen from 0, to −20, −40 and −60 °C, obvious shifting of the signature peaks can be seen (Fig. 4c–f). Since no external load was applied during the crystallization process, the chemical bond shift is believed to be due to internal stress in the lattice structure. The internal stress can be estimated to be 0.74 MPa (detailed calculations in the Supplementary Information).

The giant reversible EUC and CUH of the cPBD can be attributed to a combination effect of entropic elasticity and crystallization/melting transition. For most semicrystalline polymers, crystallization/melting transition is the driving force for the reversible actuation, which is also true for the cPBD in the melting/crystallization zone. However, the cPBD exhibits even larger actuation at temperatures above the crystallization temperature. This unique behavior has both its molecular origin and morphology contribution. It is believed that the reversible actuation above the crystallization temperature is entropy driven due to the large percentage of C=C bonds and the mesogen-like morphology. With isolated C=C bonds in the backbone, the flexible backbone of cPBD can easily bend into different configurations, which can contribute to significant entropy reduction after stretching. This is unique since most entropy driven shape memory polymers exhibit irreversible actuation only and most semicrystalline shape memory polymers depend on crystallization/melting transition only, and thus have limited reversible actuation. The crystallization process of the stretched cPBD can store sufficient stress in terms of enthalpy increase. Therefore, the reversible EUC and CUH of the cPBD is driven by both entropy and enthalpy (Fig. S14, Supplementary Information).

Therefore, to our knowledge, in order to prepare a polymeric system with giant 2W-SME, or 2W-SME with zero tensile load or with compressive load, decent crystallinity of the material itself or high crystallinity induced by stretching, plus high entropic elasticity, should be considered. To prepare a polymeric system with performance comparable with the current system, low melting and crystallization temperatures are also required. The cPBD system may be the first 2W-SMP that exhibits expansion subjected to compressive load. It may lead to application of this polymer in many areas of study such as sealant in pavement and bridge deck, gasket in pipeline, etc.

## Conclusion

Here, we report a new two-way shape memory polymer (2W-SMP) made of crosslinked *cis* poly(1,4-butadiene) (cPBD) that can be actuated below 0 °C with giant actuation strain. To the best of our knowledge, for the first time, (1) over 150% EUC and over 110% CUH in the working temperature range of −45 to 60 °C were realized under external tensile load; (2) 17.9% CUH and 6.2% EUC under 0.05 MPa compressive load were realized in this study. It was found in this study that the giant 2W-SME of cPBD is cooperatively driven by both entropy and enthalpy. It is believed that this system may find applications in many outdoor structures and devices where actuations are triggered by natural temperature change.

## Methods

*Cis* poly(1,4-butadiene) (PBD) under trade name Budene 1208 and Budene 1280 from Goodyear Chemical (Akron, OH, USA) were used as the reagents. Their viscosities are 46 and 40 (Mooney ML 1 + 4 @ 100 °C) respectively. Their onset glass transition temperature is −104 °C. All specimens used Budene 1208 as the raw material. Budene 1280 was tested to compare the reversible actuation with Budene 1208 in the supplementary text (only appears in Fig. S6). The solvent, chloroform, from Sigma-Aldrich (St. Louis, MO, USA), was used to dissolve *cis* polybutadiene. A sticky homogeneous solution was acquired after overnight stirring. The curing agent, dicumyl peroxide (DCP), from Sigma-Aldrich, at the amount of 3 wt% of *cis* polybutadiene, was added into the solution. The DCP amount of 1 wt% or 5 wt% was also used for comparisons (Fig. S5). After well mixing of the dicumyl peroxide with *cis* polybutadiene solution, chloroform was removed either by rotavap or simple evaporation. The dried *cis* polybutadiene and dicumyl peroxide mixture was then clamped in a Teflon mold and cured for 30 min in a 150 °C oven. The crosslinked *cis* polybutadiene (cPBD) in a semi-transparent color was yielded.

Dynamic mechanical analyzer (DMA) was used to investigate the thermomechanical properties of the cPBD. The temperature sweep was acquired with multi-frequency-strain mode using Q800 DMA (TA Instruments, DE, USA). The amplitude was 15 µm, the frequency was 1 Hz, and the temperature sweep range was −60 to 80 °C. The temperature ramp rate was 3 °C min^−1^. The reversible EUC and CUH under tensile load, zero load, and compressive load, as well as irreversible actuation under tension were studied using controlled-force mode. Load and temperature were pre-programmed and the strain change can be precisely recorded. The heating and cooling rate was either 10, 5 or 1 °C min^−1^.

Differential scanning calorimeter (DSC) study was conducted using PerkinElmer DSC 4000 (MA, USA). The cPBD of about 5–10 mg was placed in an aluminum pan and scanned between either −55 to 0 °C or −15 to 20 °C with heating and cooling rates at 5 °C min^−1^. Three specimens including an as-prepared, a 100% stretched and a 200% stretched were tested. The purging rate of the nitrogen gas was 30 mL min^−1^. The second heating and the first cooling cycles are shown so that the thermal history can be eliminated from the first heating cycle. The melting temperature and the crystallization temperature are labeled in the plot.

The Raman spectroscopy of an as-prepared, a 100% stretched and a 200% stretched specimens at room temperature was performed on inVia Raman Microscope (Renishaw, Wotton-under-Edge, United Kingdom). 633 nm laser and 1800 l mm^−1^ (vis) grating were used. The exposure time was 10 s and laser power was 5%. The *in situ* Raman spectroscopy of the as-prepared specimen for a cooling cycle from 0, to −20, −40 and −60 °C was acquired on Horiba Xplora PLUS Confocal Raman Spectroscopy with Linkam stage. The specimen was hold at each desired temperature for 10 min before the acquisition of the Raman spectrum. 532 nm laser and 600 l mm^−1^ (vis) grating were used. The acquisition time was 1 s.

Fourier-transform infrared spectroscopy (FT-IR) was used to scan and compare four specimens with Nicolet 6700 FTIR Spectrometer in the scan range of 4000–600 cm^−1^ at room temperature. The specimens include PBD, PBD and DCP mixture, cPBD, and 300% stretched cPBD.

The shape memory and recovery force test under compression load was conducted using eXpert 2610 MTS (ADMET, Norwood, MA, USA). The control software was MTESTQuattro. The temperature in the thermal chamber was controlled by E5AC-T digital controller (OMRON, Japan). The cPBD was first compressed to 50% of its original length at room temperature. Then the temperature of the oven was dropped to −20 °C (above its crystallization temperature) for about 10 min to temporarily fix the shape. Upon load removal and bringing MTS clamp just in contact with the compressed cPBD specimen without applying force, the recovery force curve was recorded with increase of the temperature in the oven.

The energy conversion efficiency test was conducted on MTS in tension mode. First, the specimen was tensile programmed to 200% strain at room temperature. Then the temperature of the thermal chamber was reduced to −45 °C to fix the programmed shape. The area integration of the programming plot was the energy input for the 200% tensile specimen. To acquire a series of data points for recovery force vs. displacement plot, 20 displacement values were selected and the corresponding force values were recorded. The area integration of the recovery force vs. displacement plot was the energy output for the 200% tensile programmed specimen.

A video shown the visual demonstration of the reversible actuation (quasi 2W-SME) was included in the supplementary information. Several screen shots of the process were displayed to demonstrate the giant reversible actuation under tensile load. Regarding the setup for recording the video, the cPBD bar was confined in between two clamps. The weight of 130 g (0.18 MPa) was hung to the specimen. A thermocouple was secured adjacent to the specimen and the real-time temperature can be read from the screen. Heating and cooling were generated from hair dryer and liquid nitrogen gas, respectively. Two heating and cooling cycles were proceeded as shown in Movie S1. Screen shots with the lengths of the specimen at that moment are included. Since the demonstration was recorded in open environment and there was a reasonable distance between the specimen and the thermocouple, a slight temperature difference reasonably existed between the specimen and the reading on the thermometer. In comparison, the strain changes vs temperature rise/drop at fixed stress can be accurately read through DMA plot.

The *in situ* X-ray diffraction (XRD) was performed on a Panalytical Empyrean diffractometer by using Cu as the anode material. It scanned from 5° to 40° in a step size of 0.026° at generator voltage of 45 kV and current of 40 mA. The count time is 80 sec per step. The sample stage used in the experiment was TTK-450. For each specimen, the temperature of the chamber was first decreased from 0 °C to −20 °C, −40 °C, and −60 °C, then heated it up to −40 °C, −20 °C and 0 °C. The heating/cooling rate was 5 °C min^−1^. When reaching to the desired temperature, the system was hold at that equilibrium for 10 mins prior to XRD scan.

The as-prepared specimen cPBD was also visualized with cryogenic scanning electron microscopy (cryo-SEM) (JEOL 7600 F with Gatan Alto) using secondary electrons. The specimen surface was coated with gold for about 6 nm. The accelerating voltage was 5 kV and the working distance was 8.8 mm. The images were taken at temperatures of −60 °C, −40 °C, −20 °C, and 0 °C respectively.

## Electronic supplementary material

Supplementary Information

Movie S1

## Data Availability

All other data are available from the authors upon reasonable request.

## References

[1] Leng J, Lan X, Liu Y, Du S (2011). Shape-memory polymers and their composites: Stimulus methods and applications. Prog. Polym. Sci..

[2] Hu J, Zhu Y, Huang H, Lu J (2012). Recent advances in shape–memory polymers: Structure, mechanism, functionality, modeling and applications. Prog. Polym. Sci..

[3] Meng H, Li G (2013). A review of stimuli-responsive shape memory polymer composites. Polymer.

[4] Thomsen DL (2001). Liquid crystal elastomers with mechanical properties of a muscle. Macromolecules.

[5] Chung T, Romo-Uribe A, Mather PT (2008). Two-way reversible shape memory in a semicrystalline network. Macromolecules.

[6] Pandini S (2012). Two-way reversible shape memory behavior of crosslinked poly(ε-caprolactone). Polymer.

[7] Meng Y, Jiang J, Anthamatten M (2015). Shape actuation via internal stress-induced crystallization of dual-cure networks. ACS Macro Lett..

[8] Zotzmann J, Behl M, Hofmann D, Lendlein A (2010). Reversible triple-shape effect of polymer networks containing polypentadecalactone- and poly(ε-caprolactone)-segments. Adv. Mater..

[9] Saatchi M, Behl M, Nöchel U, Lendlein A (2015). Copolymer networks from oligo(ε-caprolactone) and n-butyl acrylate enable a reversible bidirectional shape-memory effect at human body temperature. Macromol. Rapid Commun..

[10] Stroganov V (2015). Reversible thermosensitive biodegradable polymeric actuators based on confined crystallization. Nano Lett..

[11] Lee KM, Knight PT, Chung T, Mather PT (2008). Polycaprolactone−POSS chemical/physical double networks. Macromolecules.

[12] Bai Y, Zhang X, Wang Q, Wang T (2014). A tough shape memory polymer with triple-shape memory and two-way shape memory properties. J. Mater. Chem. A.

[13] Baker RM, Henderson JH, Mather PT (2013). Shape memory poly(ε-caprolactone)-co-poly(ethylene glycol) foams with body temperature triggering and two-way actuation. J. Mater. Chem. B.

[14] Razzaq MY, Behl M, Kratz K, Lendlein A (2013). Multifunctional hybrid nanocomposites with magnetically controlled reversible shape-memory effect. Adv. Mater..

[15] Razzaq MY, Behl M, Nöchel U, Lendlein A (2014). Magnetically controlled shape-memory effects of hybrid nanocomposites from oligo(ω-pentadecalactone) and covalently integrated magnetite nanoparticles. Polymer.

[16] Xie T, Li J, Zhao Q (2014). Hidden thermoreversible actuation behavior of Nafion and its morphological origin. Macromolecules.

[17] Tippets CA (2015). Dynamic optical gratings accessed by reversible shape memory. ACS Appl. Mater. Interfaces.

[18] Zhou J (2014). Shapeshifting: Reversible shape memory in semicrystalline elastomers. Macromolecules.

[19] Turner SA, Zhou J, Sheiko SS, Ashby VS (2014). Switchable micropatterned surface topographies mediated by reversible shape memory. ACS Appl. Mater. Interfaces.

[20] Li J, Rodgers WR, Xie T (2011). Semi-crystalline two-way shape memory elastomer. Polymer.

[21] Behl M, Kratz K, Noechel U, Sauter T, Lendlein A (2013). Temperature-memory polymer actuators. P. Natl. Acad Sci..

[22] Seok Jin H, Woong-Ryeol Y, Ji Ho Y (2010). Two-way shape memory behavior of shape memory polyurethanes with a bias load. Smart Mater. Struct..

[23] Raquez J-M (2011). Design of cross-linked semicrystalline poly(ε-caprolactone)-based networks with one-way and two-way shape-memory properties through diels–alder reactions. Chem. Eur. J..

[24] Bothe M, Pretsch T (2012). Two-way shape changes of a shape-memory poly(ester urethane). Macromol. Chem. Phys..

[25] Behl M, Kratz K, Zotzmann J, Nöchel U, Lendlein A (2013). Reversible bidirectional shape-memory polymers. Adv. Mater..

[26] Qi G, Kristofer KW, Patrick TM, Martin LD, Qi HJ (2013). Thermomechanical behavior of a two-way shape memory composite actuator. Smart Mater. Struct..

[27] Haberl JM (2014). Light-controlled actuation, transduction, and modulation of magnetic strength in polymer nanocomposites. Adv. Funct. Mater..

[28] Wang Z, Song W, Ke L, Wang Y (2012). Shape memory polymer composite structures with two-way shape memory effects. Mater. Lett..

[29] Qin H, Mather PT (2009). Combined one-way and two-way shape memory in a glass-forming nematic network. Macromolecules.

[30] Ma L (2015). Effects of carbon black nanoparticles on two-way reversible shape memory in crosslinked polyethylene. Polymer.

[31] Wu Y (2014). Two-way shape memory polymer with “switch-spring” composition by interpenetrating polymer network. J. Mater. Chem. A.

[32] Li W, Liu Y, Leng J (2014). Shape memory polymer nanocomposite with multi-stimuli response and two-way reversible shape memory behavior. RSC Adv..

[33] Li, G. Self-Healing Composites: Shape Memory Polymer Based Structures. *John Wiley & Sons, Inc., West Sussex, UK* (2014).

[34] Yang Q, Fan J, Li G (2016). Artificial muscles made of chiral two-way shape memory polymer fibers. Appl. Phys. Lett..

[35] Bothe M, Pretsch T (2013). Bidirectional actuation of a thermoplastic polyurethane elastomer. J. Mater. Chem. A.

[36] Li G, Li Y, Metcalf JB, Pang S-S (1999). Elastic Modulus Prediction of Asphalt Concrete. J. Mater. Civil Eng..

[37] Li G, Zhao Y, Pang SS, Huang W (1998). Experimental Study of Cement-Asphalt Emulsion Composite 11Communicated by F.W. Locher. Cem. Concr. Res.

