# Supplementary material for: Giant reversible elongation upon cooling and contraction upon heating for a crosslinked cis poly(1,4-butadiene) system at temperatures below zero Celsius
Source: Sci Rep. 2018 Sep 24;8:14233. doi: 10.1038/s41598-018-32436-9 (PMC6155190; doi:10.1038/s41598-018-32436-9)
Supplement: Supplementary file 1 — Supplementary Information [file 41598_2018_32436_MOESM1_ESM.pdf]

Supplementary Information for

Giant reversible elongation upon cooling and contraction upon heating for a crosslinked *cis* poly(1,4-butadiene) system at temperatures below zero Celsius

Lu Lu, Jinbao Cao, Guoqiang Li

This file includes:
Figures S1-18
Tables S1-5
Supplementary text
Movie S1 Caption
References

Other Supplementary Materials for this manuscript include the following:
Movie S1 (.mov format)

1. Dynamic Mechanical Analyzer (DMA) temperature scan

A temperature scan was firstly acquired using DMA (Figure S1). The changes of storage modulus, loss modulus and tan delta with temperature were recorded. At temperature -40 °C, the modulus of the material was 165 MPa, and decreased to 66.3 MPa at 0 °C and 1.5 MPa at 15 °C. The reason is believed to be the melting of crystals from -40 °C to 15 °C. Above 15 °C, the storage modulus was stabilized at about 1.5 MPa.

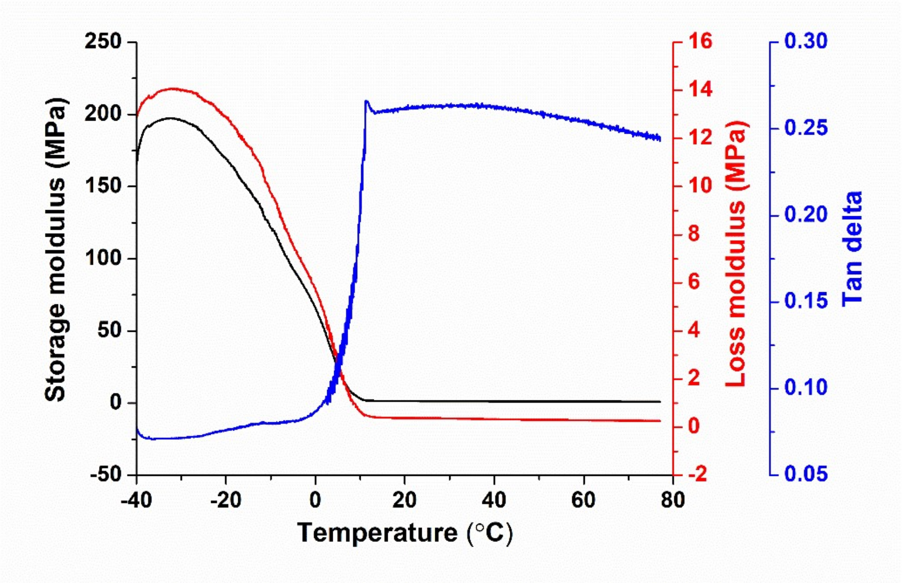

Figure S1. Temperature scan of 3 wt% DCP crosslinked *cis* polybutadiene using DMA.

2. Differential Scanning Calorimeter (DSC) scan

Due to the semi-crystalline nature of the crosslinked specimen, differential scanning calorimeter (DSC) study was conducted to locate the melting peak and the crystallization peak of specimens with varying pre-strains (Figure S2). They are as-prepared, 100% tensile stretched and 200% tensile stretched specimens. The specimens were scanned from -55 °C to 0 °C (Figure S2a). Above 0 °C, no peaks can be detected. The melting transitions of the three specimens are roughly at the same location, while the crystallization temperatures have a clear increase with increase in the pre-stretch, which agrees with the in situ XRD results (Figure 3). The enthalpy changes (ΔH) of the endothermic peaks are labeled in the plot. The higher the pre-stretch, the more the energy is needed for the melting transition. The enthalpy changes were 30.3, 32.2 and 34.5 J g^{-1} for as-prepared, 100% stretched and 200% stretched specimens, respectively. It indicates that the stored enthalpy is 1.9 J g^{-1} (32.2 – 30.3 = 1.9) for 100% stretched specimen and 4.2 J g^{-1} (34.5 – 30.3 = 4.2) for 200% stretched specimen during tensile programming.

An as-prepared cPBD and a 200% tensile stretched cPBD specimens were also scanned in the temperature range of -15 to 20 °C by DSC to avoid its crystallization zone (Figure S2b). Of course there is no crystallization transition observed for both specimens. However, a broad melting transition appeared for the 200% stretched specimen with the melting peak at -7 °C, indicating the existence of quasi-crystals or mesogens with cPBD after 200% tensile programming.

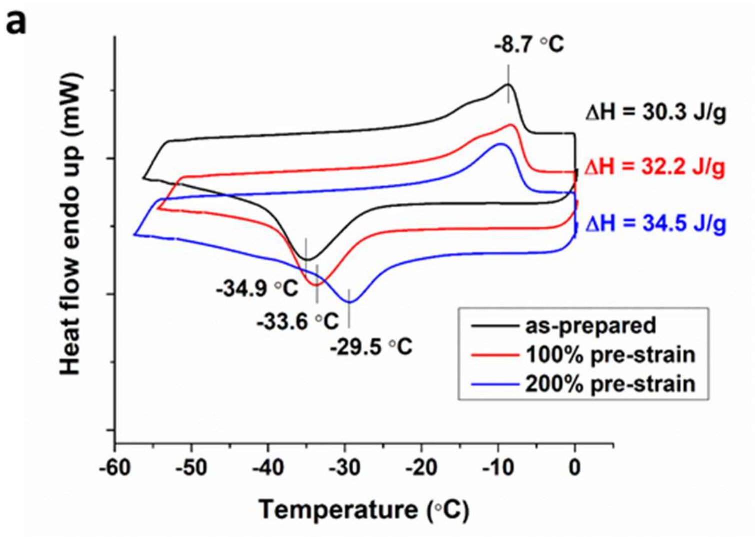

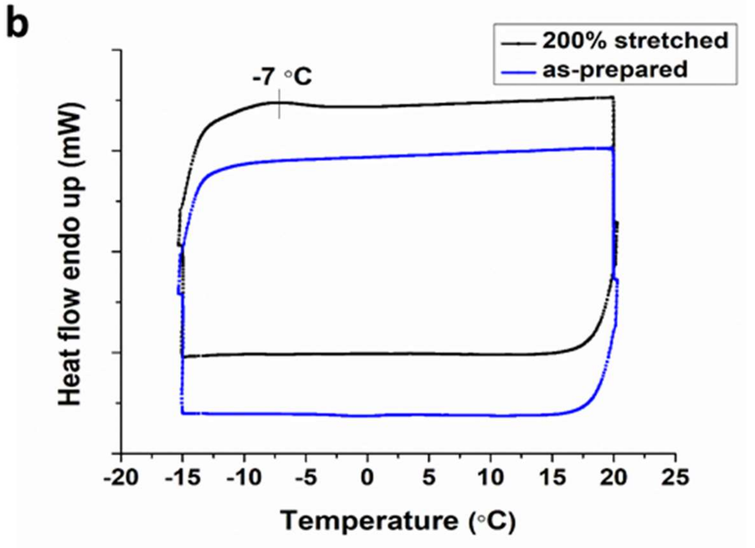

Figure S2. DSC scans of specimens with varying pre-strains within different temperature windows. (**a**) -55 to 0 °C. (**b**) -15 to 20 °C.

3. Raman spectroscopy

Raman spectroscopy is able to detect the chemical bond length change, thus it is used to validate whether tensile programming can cause any bond length change or not. If the bond length is changed, the input energy to the specimen can be stored in the form of enthalpy increase. If the bond length is not changed, the energy is stored by the entropy decrease. Three specimens of an as-prepared, a 100% tension stretched and a 200% tension stretched were scanned by Raman spectroscopy at room temperature first (Figure 4a). No clear difference was noticed among the three spectra. Signature peaks show up for all three spectra, including C=C stretching at 1652 cm^{-1}, =C-H stretching at 3010 cm^{-1}, CH_2 asymmetric stretching at 2900 cm^{-1}, CH_2 stretching at 2852 cm^{-1}, C-C stretching at 990 cm^{-1}, and CH_2 in-plane deformation at 1262 cm^{-1}. Since the test was at room temperature, the cPBD was at its molten state. No chemical bond or structure change were involved within the specimens. In other words, the energy is stored in the form of entropy decrease.

In situ Raman spectroscopy during a cooling cycle was then conducted to monitor whether there was any peak shifting or bond length changing during crystallization. The as-prepared specimen was subjected to the test. The specimen temperature was dropped from 0, to -20, -40 and -60 °C and obvious shifting of the signature peaks could be seen in Figure 4c-f. Since no external load was applied during the crystallization process, the chemical bond shift is believed to be due to internal stress. The internal stress can be estimated based on Equation S1 from a recent work:[1]

$$\sigma_{bond} = \frac{E}{1-\nu} \times \left(\frac{\Delta\omega}{\omega_0} \times \frac{N_{C=C}}{N_{total}} + \frac{\Delta\omega}{\omega_0} \times \frac{N_{=C-H}}{N_{total}} + \frac{\Delta\omega}{\omega_0} \times \frac{N_{-C-H}}{N_{total}} + \frac{\Delta\omega}{\omega_0} \times \frac{N_{C-C}}{N_{total}}\right) \quad \text{Equation (S1)}$$

where σ is the residual stress, E is the modulus, ν is the Poisson's ratio, Δω is the variation of the Raman shift, and ω_0 is the reference Raman peak (at 0 °C before crystallization). The Poisson's ratio is 0.499 for *cis* polybutadiene and the modulus is 165 MPa at -40 °C (Figure S1). The Raman shift (Δω) and the reference peak position (ω_0) can be seen in Figure 4c-f and the values are summarized in Table S1. The numbers of each bond in the repeating unit are: $N_{C=C}$: 1; N_{-C-H}: 4; $N_{=C-H}$: 2, N_{C-C}: 3; N_{total}: 10. After plugging the values into Equation S1, the internal residual stress is 0.74 MPa for the as-prepared specimen during crystallization. It indicates enthalpy increases during crystallization process, which is also validaed by the DSC test resutls in Figure S2. Although in situ Raman for the streched specimens were not conducted, it is believed that more stress can be stored in the stretched lattice, leading to increase in enthapy.

Table S1. Chemical bond shift during cooling based on Raman spectroscopy

Bond type	ω_0 (cm^{-1})	ω_{final} (cm^{-1})	Δω (cm^{-1})
C=C	1651.51	1657.35	5.84
=C-H	3007.15	3002.22	4.93
-C-H	1270.11	1264.01	6.10
C-C	991.52	985.22	6.30

4. FT-IR

Room temperature FT-IR was used to compare the infrared absorption of four specimens (Figure S3). They are PBD raw material, the mixture of PBD and DCP, cPBD, and 300% tensile stretched specimen. Signature peaks showed up for all spectra, including =C-H stretching at 3004 cm^{-1}, CH_2 asymmetric stretching at 2939 and 2849 cm^{-1}, C=C stretching at 1655 cm^{-1}, and CH_2 in-plane deformation at 1449 cm^{-1}. No clear difference among the four spectra was noticed. It indicates that upon crosslinking or tensile stretching, no obvious changes happened to the signature chemical bonds. Furthermore, because no clear signal reduction of the C=C bond signal at 1655 cm^{-1} can be seen, it can be concluded that chemical crosslinking using DCP mainly occurred at C-C bond and the main chain bending flexibility and more free volume of PBD can be retained after crosslinking, which is the molecular basis for the reversible actuation above the crystallization temperature.

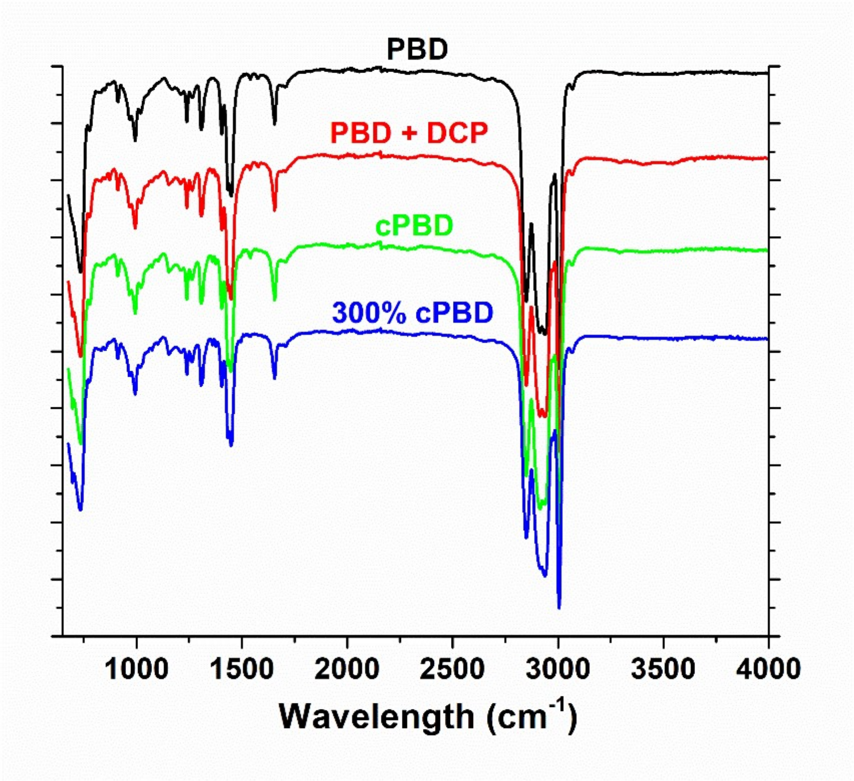

Figure S3. FT-IR spectrum of as-prepared and 300% stretched specimens.

5. The two-way shape memory effect (2W-SME) test

Usually, within a reasonable load range, the larger the external load, the bigger the 2W-SME, especially for the elongation upon cooling (EUC). In Figure S4, the external load was tuned to study the influence of loading to the 2W-SME. In Figure S4a, when ramping the load from 0.15 to 0.18 MPa, the reversible strain actuation (red curve) has a clear increase. At 0.18 MPa, the EUC was ~50% and contraction upon heating (CUH) was ~42%. The creep effect was 8% on average after one thermomechanical cycle. In Figure S4b, the load ramped from 0.24 to 0.32 MPa. At 0.32 MPa, the EUC was ~93% and CUH was ~76%. The creep effect was 9% on average after one thermomechanical cycle. This test clearly showed that tuning the external tensile load during experiment is an efficient way to tune the strain actuation level.

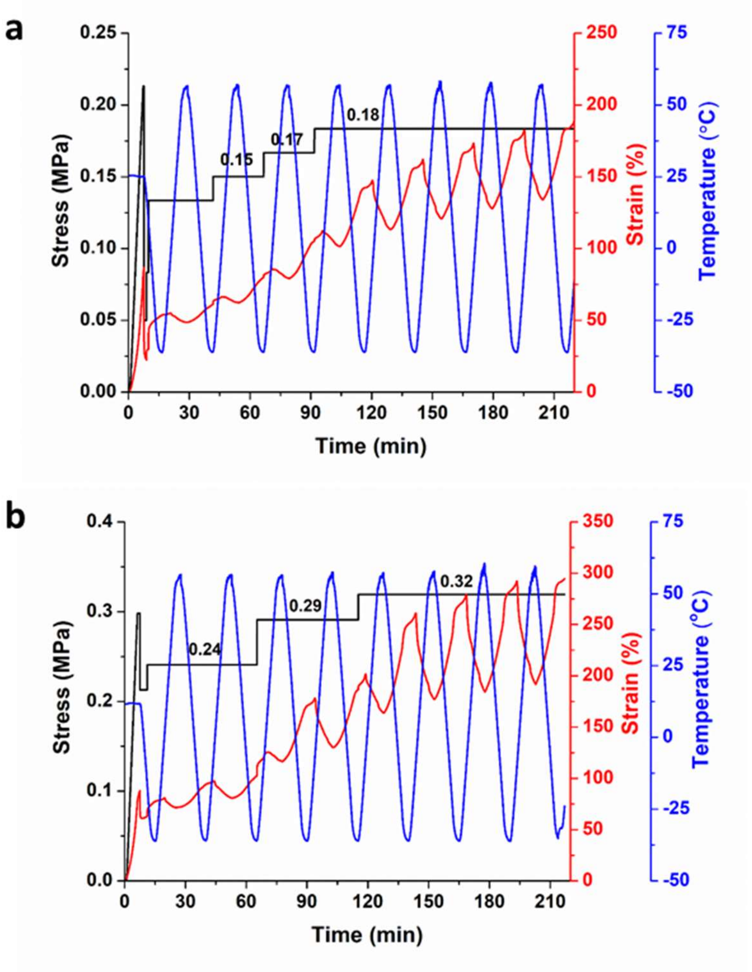

Figure S4. The two-way shape memory effect (2W-SME) of cPBD under different external tensile load. (**a**) Increase tensile load from 0.15 to 0.18 MPa. (**b**) Increase tensile load from 0.24 to 0.32 MPa.

The influence of crosslinking level on the 2W-SME was investigated (Figure S5). When curing *cis* polybutadiene with only 1 wt% DCP, the network was not stable enough (Figure S5a). At 0.014 MPa load, EUC was observed but no CUH. At 0.008 MPa load, no CUH was noticed, either. When curing with 3 wt% DCP, the EUC was ~50% and CUH was ~42% under 0.18 MPa load (Figure S5b). When curing with 5 wt% DCP, the EUC was ~42% and CUH was ~36% under 0.25 MPa load (Figure S5c). Due to the better two-way shape memory performance with smaller load when comparing systems cured with 3 wt% DCP to 5 wt% DCP and the cost effectiveness of using less curing agent, 3 wt% DCP was considered the best curing condition within these three.

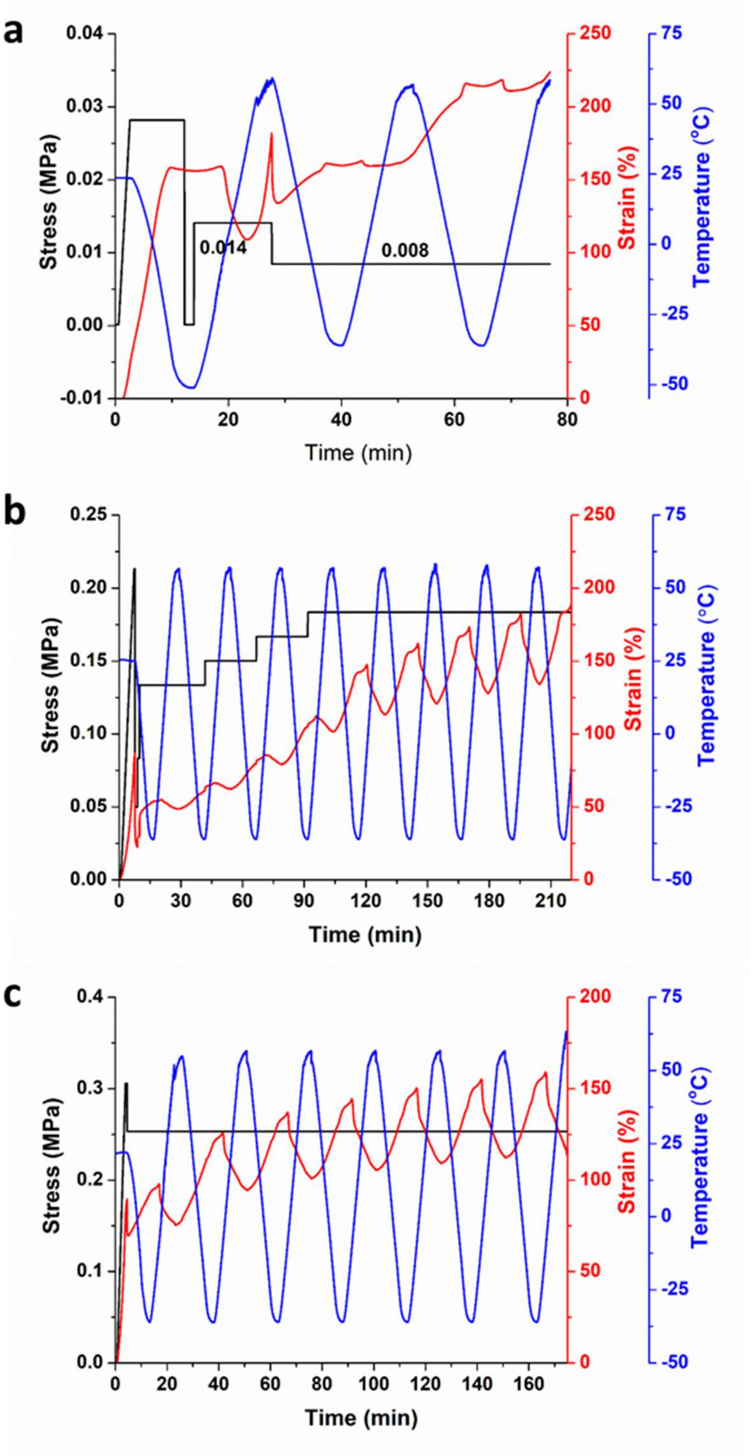

Figure S5. The 2W-SME of cPBD cured with different amount of DCP. (**a**) 1 wt%. (**b**) 3 wt%. (**c**) 5 wt%.

The *cis* polybutadiene under trade name Budene 1208 was used throughout this work. Its viscosity is 46 (Mooney ML 1+4 @ 100 °C) and its *cis* content is 97%. To study the molecular weight or viscosity influence on the 2W-SME, another raw material - *cis* polybutadiene under trade name Budene 1280 was also investigated (Figure S6a). The viscosity of Budene 1280 is 40 (Mooney ML 1+4 @ 100 °C) and its *cis* content is also 97%. For cPBD prepared with Budene 1280, the EUC is ~38% and the CUH is ~11% under 0.02 MPa load (Figure S6a). The creep effect was 23% on average after one thermomechanical cycle. For cPBD prepared with Budene 1208, the EUC is ~50% and the CUH was ~42% under 0.18 MPa load (Figure S6b). The creep effect was 8% on average after one thermomechanical cycle. It can be seen that with higher viscosity/molecular weight for Budene 1208, a more stable polymer network and better 2W-SME can be obtained.

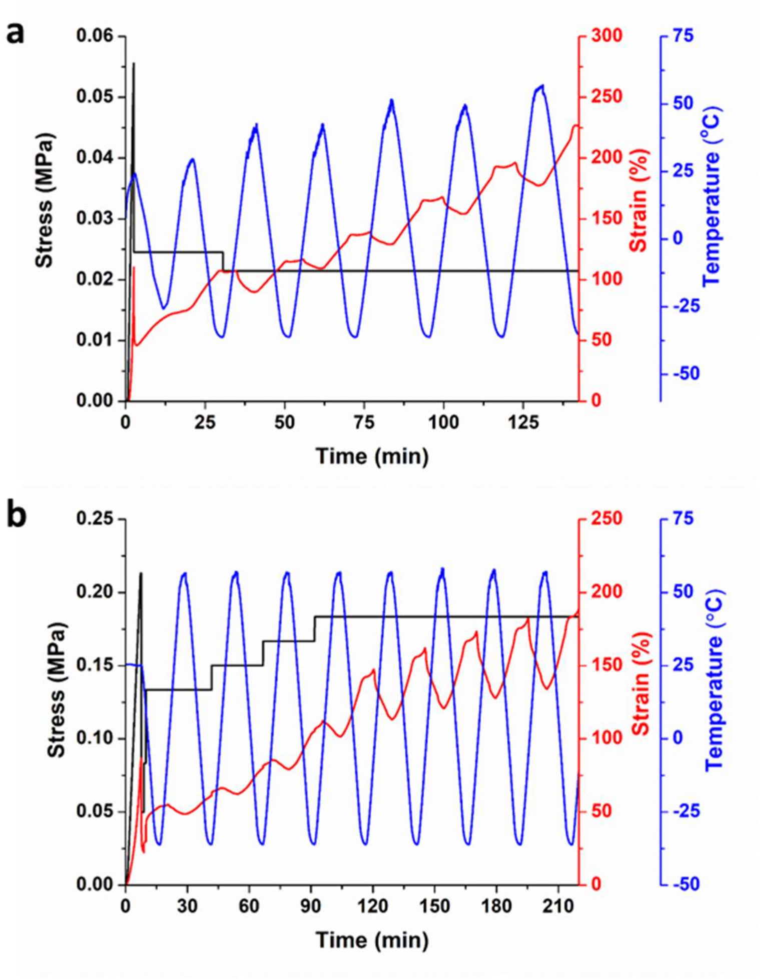

Figure S6. The 2W-SME of cPBD prepared with different raw materials. (**a**) Budene 1280. (**b**) Budene 1208.

A video demonstrating the reversible EUC and CUH of the cPBD was recorded (Movie S1). Several screenshots are displayed in Figure S7 and the values of the strain changes are summarized in Table S2. In Figure S7a, the original length of the sample was 1.0 cm. After applying load (130 g or 0.18 MPa), the length increased to 4.2 cm (Figure S7b), which equals to 320% tensile programming strain. Then, the temperature dropping (Figure S7c, e,g) induced elongation of the specimen with strain change of 150%, 180% and 180%, respectively. The temperature rising (Figure S7d,f,h) induced contraction of the sample with strain change of 130%, 170% and 180%, respectively. At the end, the length of the

specimen was 1.4 cm after the load removal (Figure S7i). When compared to the original specimen length of 1.0 cm, the sample creeped 40% after 5 heating and cooling cycles (8% each cycle).

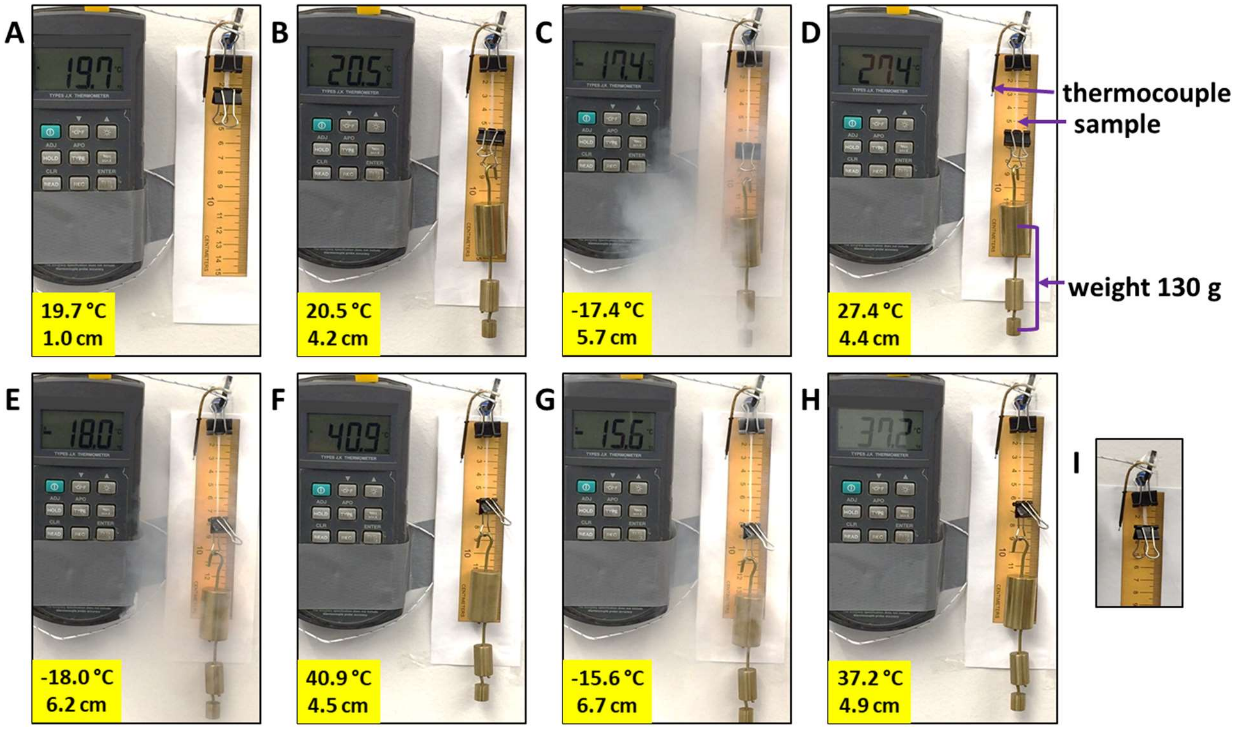

Figure S7. Screenshots of the reversible EUC and CUH of the cPBD with temperature change. (**a**) Initial state. (**b**) Adding 130 g load. (**c**) Cooling to -17.4 °C. (**d**) Heating to 27.4 °C. (**e**) Cooling to -18.0 °C. (**f**) Heating to 40.9 °C. (**g**) Cooling to -15.6 °C. (**h**) Heating to 37.2 °C. (**i**) Returning to room temperature and load removal.

Table S2. Length of the specimen at different stage in Figure S7 and calculations of the strain change.

	start point	add load	cool	heat	cool	heat	cool	heat	remove load
length (cm)	1	4.2	5.7	4.4	6.2	4.5	6.7	4.9	1.4
Δε (%)		320	150	130	180	170	180	180	40

6. Coefficient of Thermal Expansion (CTE)

Some materials can demonstrate negative coefficient of thermal expansion (NCTE), i.e., expansion upon cooling and contraction upon heating, which is similar to the 2W-SME discussed here. Therefore, it is necessary to make sure that the reversible actuation of the cPBD is not due to NCTE. In Figure 2c, zero external load and zero pre-strain (i.e., without programming) were applied in the experiment. It is shown that the specimen has positive CTE behavior, which expands upon heating and contracts upon cooling. Within temperature window -45 to 0 °C, 0.9% expansion upon heating and 0.8% contraction upon cooling were visualized. Within temperature window -45 to 55 °C, 1.8% expansion upon heating and 1.4% contraction upon cooling were observed. The slight difference between cooling and heating branches was attributed to the small creep of the polymer under gravitational force during heating. It indicates that the reversible EUC and CUH do not come from the NCTE and programming is the requirement for the specimen to display reversible actuation.

Careful examination of Figure 2c shows that for each cooling or heating branch, there is a clear transition point with different slope or CTE. This result again validates that the cPBD can be divided into a high temperature zone and a low temperature zone, and their behavior (reversible actuation, CTE, etc.) is controlled by different mechanisms and thus is different.

7. The two-way shape memory effect (2W-SME) of the cPBD within different temperature windows

The width of the working temperature window influences the reversible actuation of the cPBD as demonstrated in Figure 1. An additional test is shown in Figure S8. The lowest working temperature for each cycle is the same, which is -50 °C, while changing the highest temperature (T_{high}) for each cycle. The external tensile load is 0.22 MPa throughout the test. All essential data of experimental conditions and results are summarized in the table above the figure. When gradually widening the temperature window, it can be seen that the CUH and EUC were clearly increased. When looking carefully, the slope of most cooling cycles have a turning point, except for the first two cycles whose EUCs were too small to detect this change. The slope increased after the turning point and three points were randomly selected and labeled with green arrows for the readers to locate the points. The data were analyzed further by revealing the turning temperature (T_{turn}) for each cooling cycles, EUC before T_{turn} and EUC after T_{turn}. It turns out that all turning temperatures are around the crystallization temperature of the cured specimen. The EUC below T_{turn} has a smaller increase while EUC above T_{turn} has a larger increase with widening of the temperature window. The reason is that the EUC below T_{turn} is due to crystallization, which is only influenced by the amount of crystals melted in the previous heating cycle because the lowest temperature and external load are the same for each cycle. However, the EUC above T_{turn} is due to entropy elasticity which is greatly influenced by how wide the temperature window is.

T_{high} (°C)	-2	8	20	30	40	50	66
CUH (%)	4	31	35	41	51	62	75
EUC (%)	7	33	40	51	63	79	111
T_{turn} (°C)	N/A	-27	-32	-32	-31	-27	-27
EUC above T_{turn} (%)	N/A	17	22	32	39	55	84
EUC below T_{turn} (%)	N/A	16	18	19	24	24	27

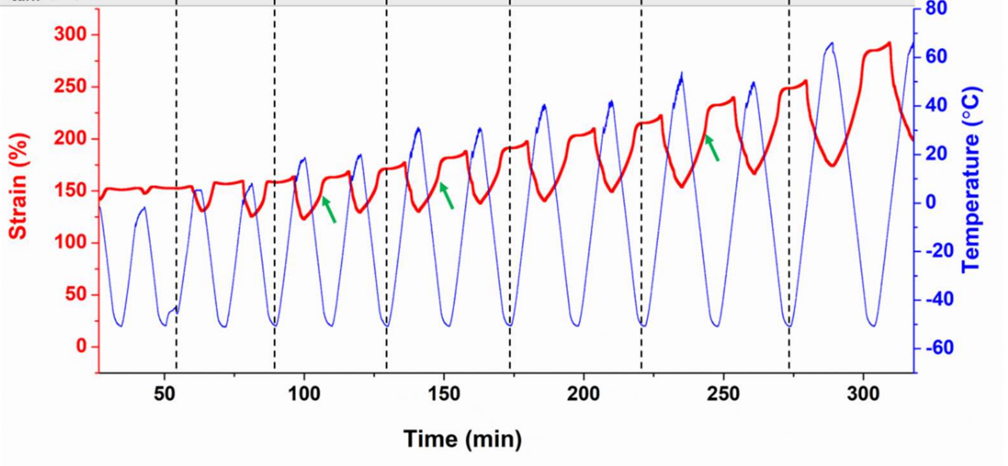

Figure S8. The 2W-SME study of cPBD within different temperature windows.

8. The 2W-SME without External Tensile Load

The two-way shape memory effect (2W-SME) under zero external load during actuation was explored (Figure S9). A load of 0.22 MPa was applied to the specimen and it was then cooled to -45 °C. The specimen was fixed to 123% strain level after the programming. Then, it was unloaded to zero stress. After that, the temperatures were swept within -50 to -5 °C, -50 to -3 °C, and -50 to -1 °C, which did not induce EUC and CUH. When expanding the temperature window to -50 to 1 °C, reversible actuation with 28% CUH and 5.3% EUC were achieved. Further increasing the upper limit of the temperature window to 3 °C leads to 34% CUH and 1.3% EUC. Therefore, it is clear that the cPBD has reversible actuation without the external tensile load within certain temperature windows, as long as the upper temperature limit is able to induce partial crystal melting but not complete shape recovery.

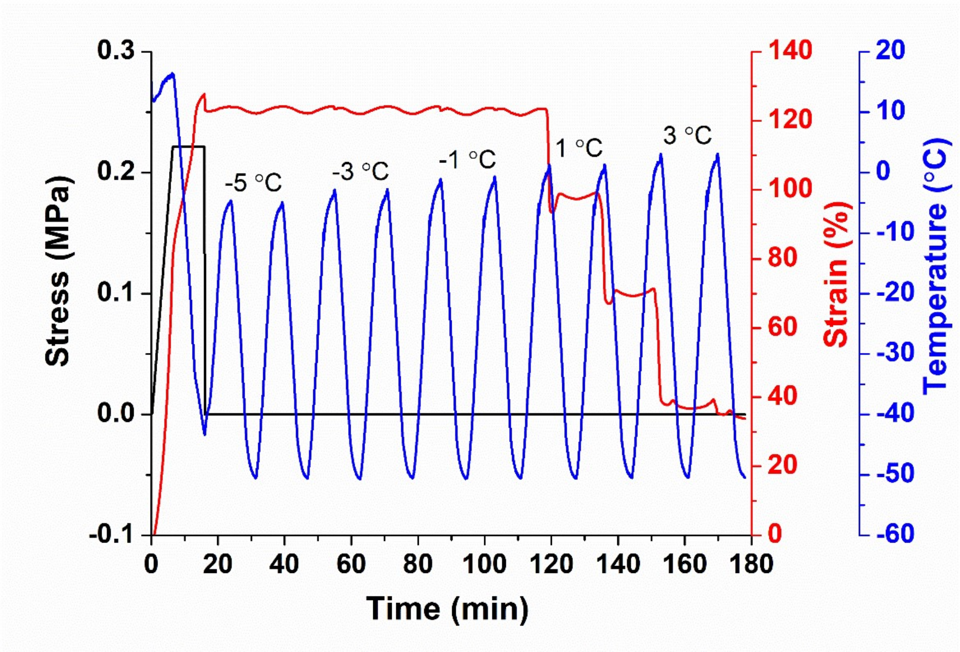

Figure S9. The 2W-SME without the external tensile load of the crosslinked Budene 1208.

9. Effect of buckling and compressive strain on CUH for the reversible actuation under external compressive load

The reversible actuation subjected to external compressive load has been reported in Figure 2. It shows clear expansion under compressive load upon cooling. For CUH, we need to examine whether or not it is caused by the buckling of the specimen or compressive strain from the compressive load.

Based on Euler's critical load equation, the maximum load which a column can bear without buckling is given by:

$$P_{cr} = \frac{\pi^2 EI}{(KL)^2} \quad \text{Equation (S2)}$$

where

P_{cr} is Euler's critical load or axial compression load on column,

E is modulus of elasticity of the column material, which is 165 MPa based on DMA temperature scan for our specimen,

I is minimum area moment of inertia of the cross section of the column, which is $\frac{1}{12} \times 2.88 \times 1.48^3 = 0.78$ mm^3,

L is unsupported length of the column, which is 12 mm on average in our experiment,
K is column effective length factor, which is 0.5.

Therefore the value of P_{cr} is 35 N. However, in the experiment, the largest compressive force used was only 0.5 N. Therefore, no buckling event would occur. Even using the modulus of 66.3 MPa at 0 °C, the buckling load is 14 N, which is still much higher than the applied load of 0.5 N. Therefore, buckling is ruled out.

Moreover, the highest compressive strain could be generated in the experiment by the compressive load at -40 °C is

$$\frac{0.12\ MPa}{165\ MPa} \times 100\% \ = 0.073\%$$

However, the CUH observed in the experiments is in the range of 2.6% to 20.3%, which is much larger than 0.073%. Again, even at 0 °C, the compressive strain is 0.18%, which is still negligible. Therefore, the contribution of compressive load induced compressive strain to CUH can be ignored.

10. The one-way shape memory effect of the cPBD

One-way shape memory effect (1W-SME) of the cPBD was investigated to better understand the cPBD (Figure S10 and Table S3). Three temperatures 60 °C (Figure S10a), 20 °C (Figure S10b), and -20 °C (Figure S10c), which are above the crystallization transition, and one temperature -45 °C (Figure S10d), which is below the crystallization transition were used for programming. An optimized tensile load (0.21 MPa) was added to the specimen at each temperature for 120 min, and then the load was removed. The specimen was then allowed to have a free shape recovery at 20 °C. The lengths, programmed strains, and recovery ratios are listed in Table S3. It can be seen that under the same load for 120 min, the higher the programming temperature, the higher the programming strain and fixed strain. The recovery ratios for specimens programmed at 60, 20 and -20 °C are fairly good for over 90%, indicating that cPBD has great 1W-SME performance at its amorphous state. When programming the specimen at -45 °C, well below its crystallization temperature, the specimen was able to be tensile programmed to only 6% strain under 0.21 MPa load. After unloading and fixing the programming strain at -45 °C, the specimen was slowly heated up at 0.5 °C min^{-1} to -3.5 °C, which expanded 1.5%, obviously due to the positive CTE effect. Continuously heating the specimen from -3.5 to 4.5 °C, the specimen contracts 4.5% due to crystal melting or shape recovery. Then, heating to room temperature induced ~2.5% expansion, again due to the positive CTE effect, not 1W-SME.

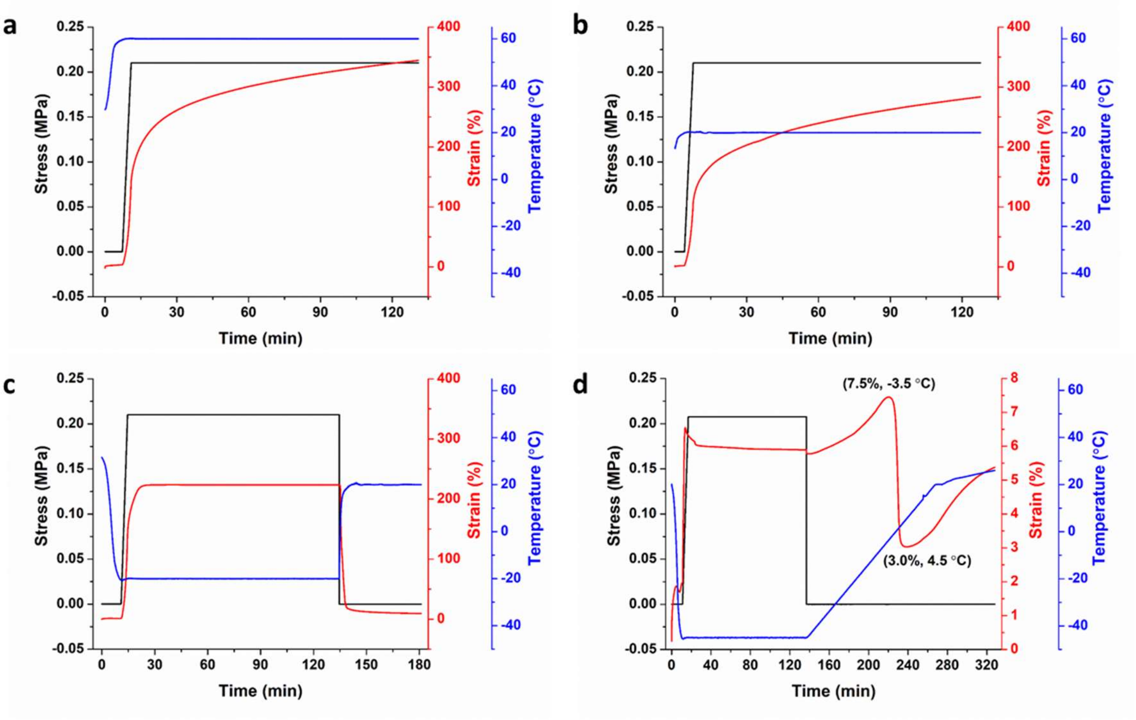

Figure S10. The one-way shape memory effect study of the cPBD under four programming temperatures. (**a**) 60 °C. (**b**) 20 °C. (**c**) -20 °C. (**d**) -45 °C. It is noted that we did not show the unloading and free shape recovery steps for the 60 °C and 20 °C tests because they are equal to or above the shape recovery temperature (20 °C).

Table S3. Analysis of the 1W-SME tests.

Temperature (°C)	Original length (mm)	Programmed length (mm)	Recovered length (mm)	Programmed strain (%)	Recovery ratio (%)
60	6.11	23.14	7.37	345	93
20	5.04	21.98	6.51	284	91
-20	4.89	15.75	5.35	224	96

Similar to the test shown in Figure S10b, after keeping 0.21 MPa stress on the specimen at 20 °C for 120 min, the load was kept and temperature was swept at different temperature windows (Figure S11). It shows that the specimen definitely has EUC and CUH at both its amorphous state and crystallization state. This is a very interesting discovery as amorphous SMPs can only show 1W-SME, not 2W-SME. Here we show that, at amorphous state, the cPBD has both 1W-SME and 2W-SME.

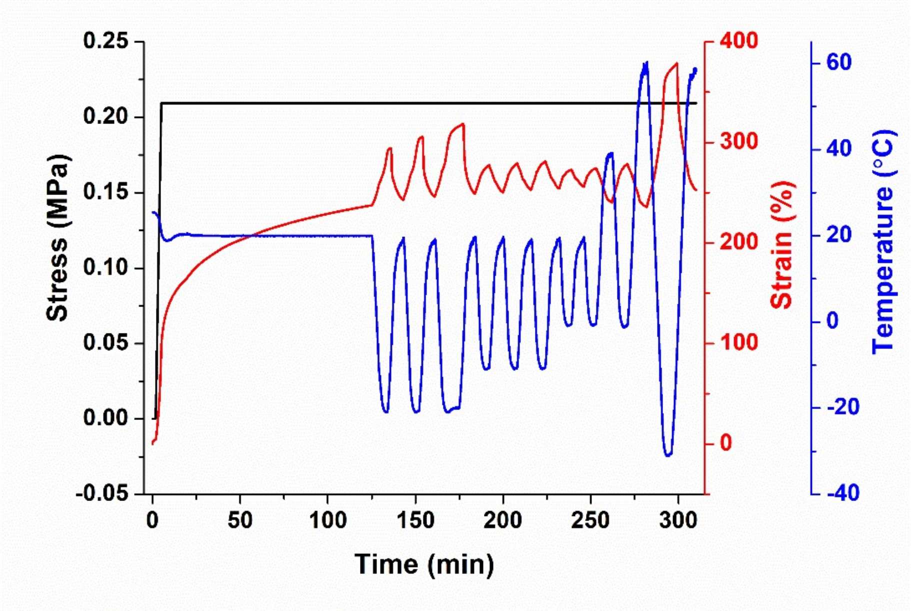

Figure S11. Reversible EUC and CUH of the cPBD after programming at 20 °C without load removal. Even when the temperature window is narrowed within the amorphous region, for example 0 to 20 °C, the cPBD still shows reversible actuation.

1W-SME under compression programming was also conducted using MTS as shown in Figure S12. The cPBD was compression programmed to 53% strain at room temperature and the compressive stress reached 0.065 MPa. Then the temperature of the MTS oven was dropped to -23 °C in 10 min to fix the compressed strain. The stress decreased to 0.0053 MPa after 10 min equilibrium. The 0.0053 MPa stress was then unloaded by lifting up the MTS clamp. The clamp was quickly brought just in contact with the compressed cPBD specimen without applying any load. The recovery force during heating to 52 °C was recorded, which is 0.011 MPa at 52 °C. It can be seen that the stress recovery was only 17% (100%×0.011/0.065) of the applied stress during programming. The shape memory effect under tension is better than compression due to directed configuration change along with the loading direction.

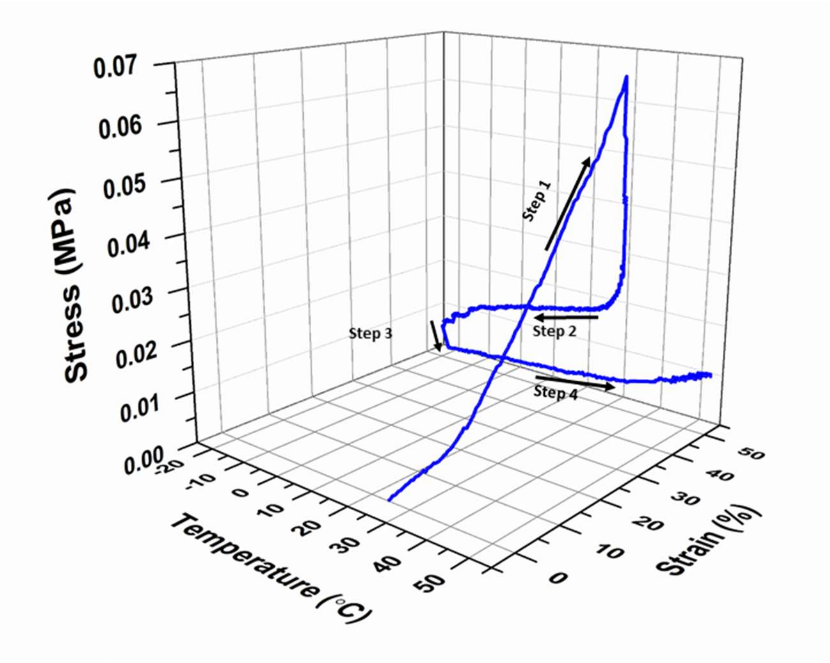

Figure S12. The one-way shape memory effect of cPBD by compression programming. This figure shows (1) compression at 25 °C, (2) cooling to -23 °C while holding the strain constant, (3) unloading at -23 °C, and (4) constrained stress recovery by heating to 54 °C.

11. Mechanical energy conversion efficiency

Energy conversion efficiency is crucial for materials who may be constructed into actuators, artificial muscles, etc. Based on thermodynamics, the cooling and heating process involves energy conversion, and the energy conversion efficiency is smaller than 100%, depending on the devices or mechanisms used for cooling and heating. Therefore, for simplicity, the mechanical energy conversion efficiency of the cPBD was calculated based on the method shown below. A specimen was tensile programmed to 200% of its original length and then cooled down to -45 °C to fix the shape. This specimen was taken as an example to calculate its energy conversion efficiency. Of course the programming level will influence the energy input and output values. The energy input can be calculated based on the area integration of the load vs. displacement plot during programming (Figure S13a), which is 32.3 mJ. The energy output value can be calculated based on the area integration of the recovery force vs. displacement plot during recovery (Figure S13b), which is 14.9 mJ. The mechanical energy conversion efficiency can be calculated as below:

$$\text{Mechanical energy conversion efficiency} = \frac{Energy\ output}{Energy\ input} \times 100\% = \frac{14.9}{32.3} \times 100\% = 46.1\%$$

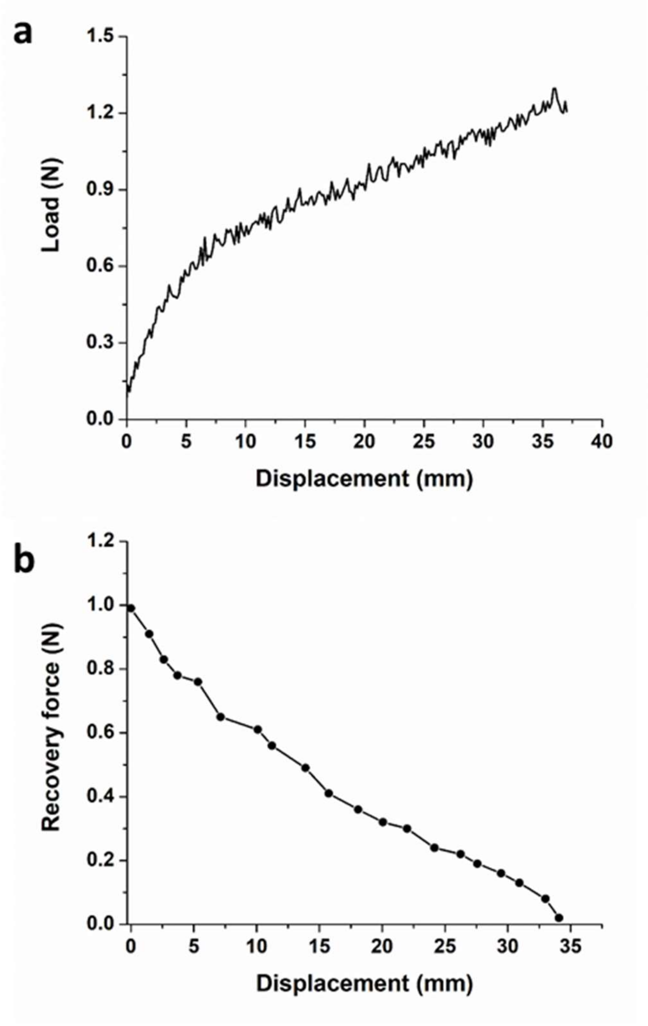

Figure S13. Energy conversion efficiency test. (**a**) Load vs. displacement profile when programming specimen to 200% strain. (**b**) For the 200% tension programmed specimen, the changes of the recovery forces at different displacements.

12. Understanding of the mechanisms

By comparing the 2W-SME results of the cPBD with previous poly(cyclooctene)[2] and polyethylene[3] studies, it is noticed that the entropic elasticity contribution to the three systems has the trend of cPBD > poly(cyclooctene) > polyethylene. It seems like increasing C=C bond content within the polymer main chain will amplify entropic elasticity contribution. As mentioned previously, cPBD has better main chain bending flexibility as compared to saturated polymer chains such as polyethylene. The good chain flexibility originates from less restricted rotation of C-C bonds adjacent to C=C bonds due to larger bond angle of 120° and less substitution groups. It contributes to more conformation possibilities thus high entropy before tensile programming, and high entropy reduction after programming. This will lead to better actuation in entropic elasticity region (high temperature zone in Figure S14). In addition, the restricted rotation of *cis* C=C bonds creates more free volume in between chains as compared to systems only with C-C bonds, which can pack chains more efficiently. This additional free volume enables better chain mobility and more configurations, and consequently large entropy change upon temperature change. These together are responsible for the better actuation of the cPBD as compared to poly(cyclooctene) and polyethylene in the entropic elasticity region.

It is believed that the crystallization/melting of the crystals within the cPBD also contributed to the reversible actuation within the crystallization/melting transition window. Based on the XRD study, the crystallinity of cPBD can increase from 13% to 68% with a 200% tensile programming. In a reversible actuation cycle, the thermal energy input/output from temperature increase/decrease will be canceled out. This leaves the programing mechanical energy the only energy to be stored in the cPBD specimen via entropy decrease and the residual stress within crystals. The stored energy is responsible for the reversible actuation without the external tensile load or even with external compressive load. For a 200% tensile stretched specimen, 2.21 MPa residual stress can be stored within the crystals upon crystallization based on XRD (Figure 3 and Table 1). For as-prepared specimen, in situ Raman spectroscopy during a cooling cycle was conducted (Figure 4). The internal residual stress upon crystallization is estimated to be 0.74 MPa.

Hence, based on our understanding, the reversible actuation of the cPBD can be separated into a high temperature zone and a low temperature zone (Figure S14). In the high temperature zone (above the crystallization temperature of cPBD), the specimen exhibits both quasi 2W-SME (Figure 1d) and 1W-SME (Figure S10a-c and Figure S11), i.e., quasi 2W-SME if the external tensile load is maintained during heating, or 1W-SME if the external tensile road is removed during heating. The specimen becomes quasi-crystalline, or forms a mesogen-like phase, at temperatures above the crystallization temperature after tensile programming (Figure S2b). In the low temperature zone, reversible actuation under external tensile load (quasi 2W-SME), without any load (true 2W-SME), or even with external compressive load (advanced 2W-SME) can be achieved. For the quasi 2W-SME, upon cooling, positive CTE leads to a slight contraction while crystallization leads to an obvious elongation. The overall behavior of the specimen upon cooling is elongation. The differences between reversible actuation with external tensile load and external compressive load are: 1) the reversible actuation with external tensile load is much bigger since the specimen is under tensile load; 2) reversible actuation under tensile load can be considered as truly reversible with only a tunable amount of creep effect, while reversible actuation under compressive load can only be regarded as semi-reversible, which means that there is always a partial expansion upon cooling after the contraction upon heating (Figure 2). In summary, the cooling induced expansion of cPBD is driven by both entropy decrease (high temperature zone) and enthalpy increase (low temperature zone in terms of storage of residual stress), while the heating induced contraction is driven by the stored tensile stress in terms of enthalpy release (low temperature zone) and entropy increase (high temperature zone). The driving force is stored in the aligned molecules (mesogen phase) and lattice structures within crystals as residual stress.

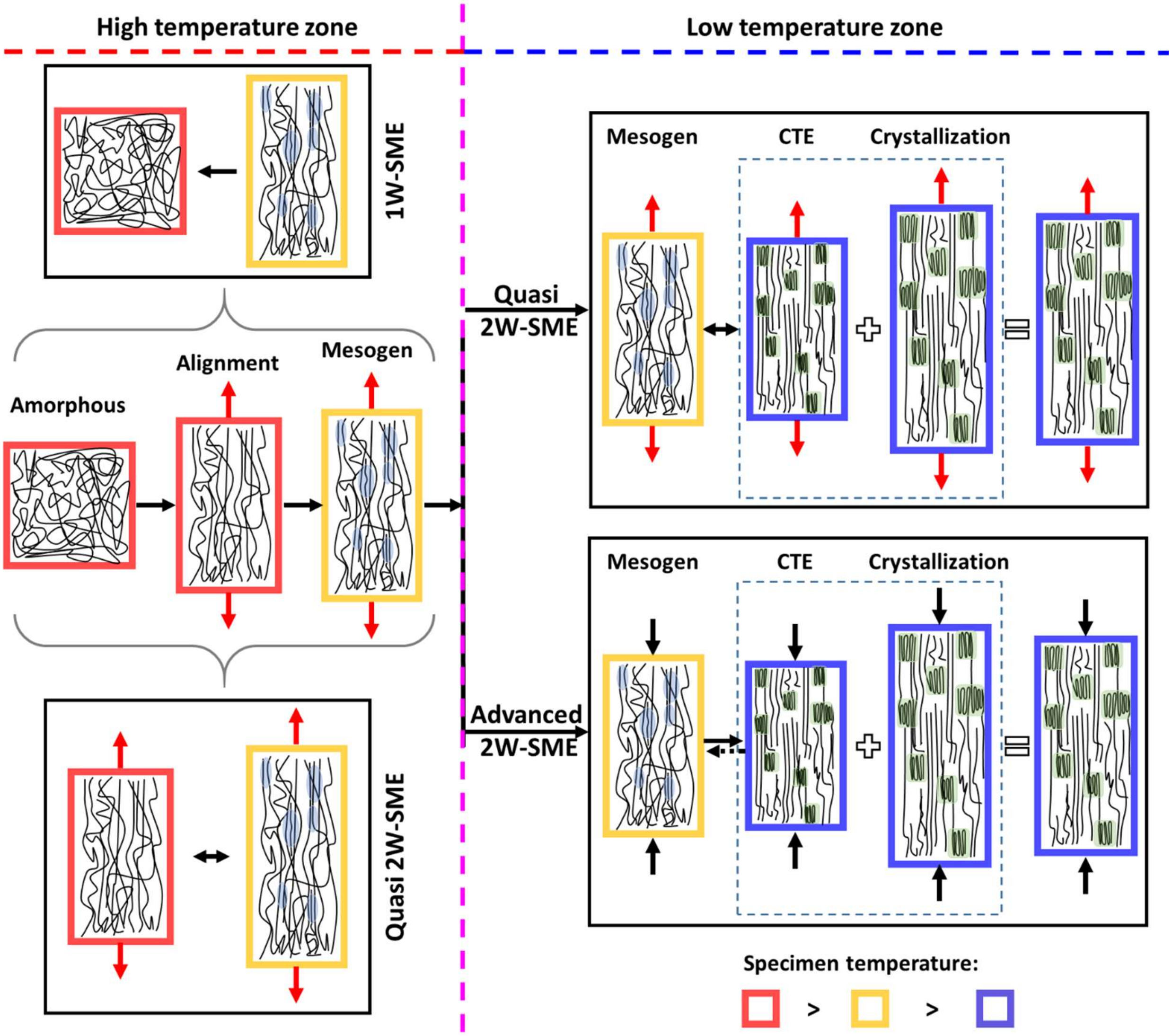

Figure S14. Proposed mechanism of quasi 2W-SME, advanced 2W-SME, and 1W-SME of the cPBD at high and low temperature zones. In the high temperature zone (above the crystallization temperature), the polymer has both 1W-SME and 2W-SME and the driving force is entropy change in terms of segments alignment, forming mesogen type of microstructures due to the large number of available configurations in the cPDB; in the low temperature zone (below the crystallization temperature), the polymer exhibits primarily 2W-SME, which is driven by enthalpy change in terms of residual stress stored in the lattice structures.

13. Mechanical and environmental durability test

In order to evaluate if the polymer can be used as sealant in pavement, which is subjected to repeated traffic load, fatigue testing is conducted. It represents the material's service life under multiple tension and compression cycles. Generally, a lower environmental temperature will shorten the time until fatigue failure. A temperature -35 °C was used for fatigue testing. The specimen was subjected to a cyclic strain of -30% to 30% for 8,500 load repetitions (Figure S15). A temporary increase in temperature (blue line) occurred every few hours, as a result of liquid nitrogen refilling. After 8,500 cycles, a slight stress decrease was observed, presumably due to structural relaxation. No other signs of fatigue failure were observed.

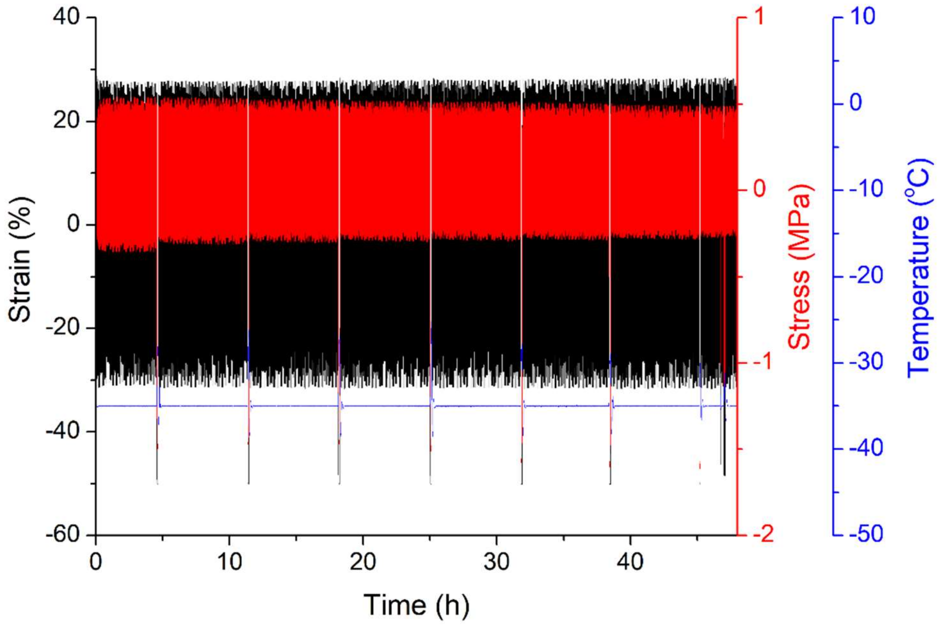

Figure S15. Fatigue testing with cyclic strain of -30% to 30% for 8,500 load repetitions.

In outdoor environment, the polymer will be subjected to moisture attacks. The response of the polymer to rainwater is important when it is used as sealant because sealants will generally be in periodic contact with rainwater. A potential loss in 2W-SME behavior after rainwater exposure could be a concern. Therefore, weight changes of two specimens immersed in rainwater for three months were monitored. Only very slight weight increases were noticed (Table S4). Then, the 2W-SME of a specimen immersed in rainwater for 67 days was tested using DMA. It turned out that rainwater had essentially no influence on the 2W-SME performance of the specimen.

Table S4. Weight changes of two cured Budene 1208 specimens in rainwater

#	Initial	20 min	5 h	50 h	100 h	12 days	46 days	90 days
Specimen 1 (mg)	91.71	91.62	91.72	91.83	91.76	92.23	92.41	92.38
Specimen 2 (mg)	100.68	100.80	100.76	100.98	100.91	101.08	101.29	101.28

14. Adhesion between the developed 2W-SMP and cement concrete

Figure S16 shows concrete blocks bonded by the 2W-SMP sealant under gravity load. It was clear that the 2W-SMP sealant can be tightly bonded to the cement concrete surface at room temperature. Because cold temperatures present a larger concern for bonding, we conducted a low temperature bonding test. In particular, we tested bonding between an asphalt based smart sealant and concrete. We used a direct tensile test configuration. We sandwiched a layer of our sealant between two concrete bars. The length of the concrete bars was 48 cm, and the cross section was a square with a side length of 2.5 cm. The sealant thickness was 0.2 cm. The choice of the dimension was to make the specimen a scaled-down version of a real pavement with a 2.5 cm wide sealant sandwiched between two 600 cm long concrete slabs. In other words, the ratio of the concrete slab length to the sealant width was the same (viz., 48 cm / 0.2 cm = 240, and 600 cm / 2.5 cm = 240). In this way, the test results can be a good representation of the real world situation. We then clamped the sandwich specimen in the MTS machine

and cooled the specimen by liquid nitrogen, with a cooling rate of 3.81°C/min. We recorded the tensile stress changes with temperature. As shown in Figure S17, the tensile stress continued to grow even at a temperature of -40°C, which is a good indication that the sealant had bonded to the concrete and the adhesion was maintained.

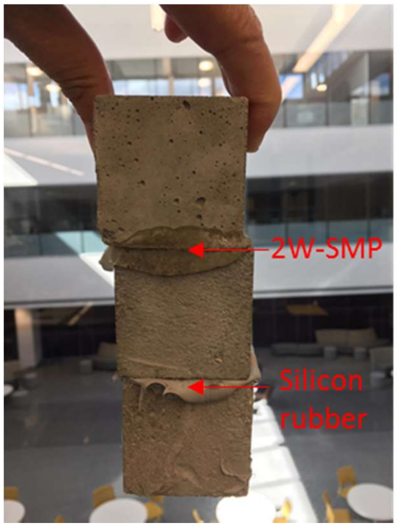

Figure S16. Concrete blocks bonded with the 2W-SMP sealant under gravity load (0.003 MPa).

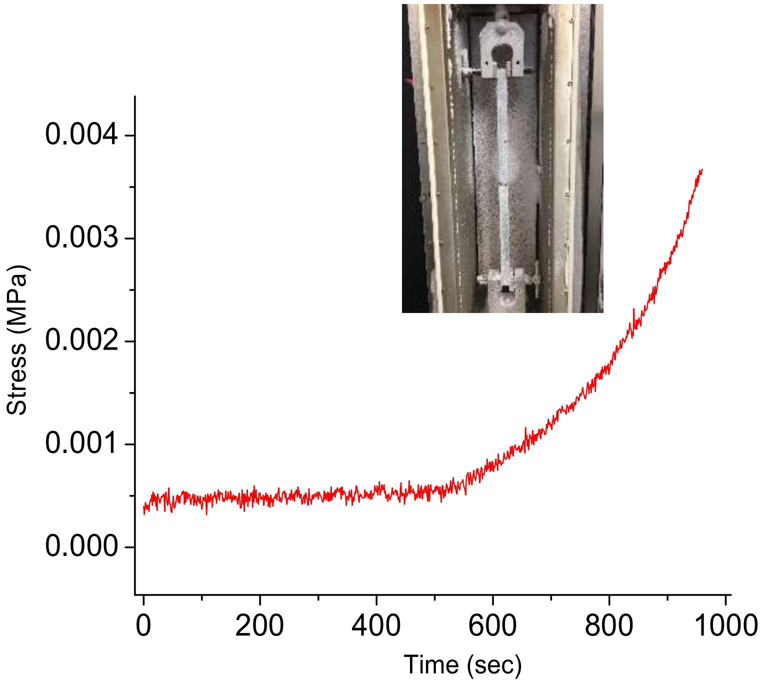

Figure S17. Development of tensile stress with temperature in the direct tension test configuration. The stress continuously grew up to -40°C, suggesting strong bonding.

15. Natural temperature drops induced elongation upon cooling (EUC)

In order to evaluate if the natural outdoor cooling can induce the EUC or not, DMA was used to simulate sealant service condition. Generally, joint sealant will be installed during Spring/Summer/Fall. When winter arrives, the temperature drop would induce gradual contraction of concrete pavement. Since the sealant adheres to the concrete slab, the contraction of concrete slabs on both sides of the sealant will apply tensile stress to the sealant, which thus has similarities to tension programming. Two types of tests were conducted.

In Fig. S18A, we gradually lowered the temperature, and at the same time gradually increased the tensile stress, simulating the real world cooling process of a sealant, to -45°C. After that, we kept the stress constant, but cycled the temperature. The 2W-SME was triggered. This result suggests that, after the first cooling process during the first cold front in the winter, the 2W-SMP is trained, or programmed. Subsequent cooling and heating (e.g., daily temperature changes) exhibit the 2W-SME. In other words, there is no need to program the 2W-SMP before installation. The natural cooling provides the required programming.

In Fig. S18B, we simulated the daily temperature cycle, again, without programming. With cooling, we gradually added tensile stress to the specimen, and with heating, we gradually removed the applied load. As shown in Fig. S18B, the temperature drop (blue line) induced a step increase in stress on the sealant (black line), and the strain reached ~150%. Temperature increase led to expansion of the concrete slab, and stress on the sealant was gradually released, resulting in contraction of the sealant. At the completion of each cooling/heating cycle, the stress became zero and the strain also became zero. While the thermal cycle accompanied by loading and unloading cannot be directly regarded as 2W-SME, because loading will cause extension and unloading will cause shortening, we do believe that the 2W-SME and the mechanical loading/unloading effect may be coupled here.

We again conclude from this study that tension programming of sealant is not required prior to installation. Natural temperature drops in the concrete slab can create tensile stress in the sealant to induce the 2W-SME of the smart sealant.

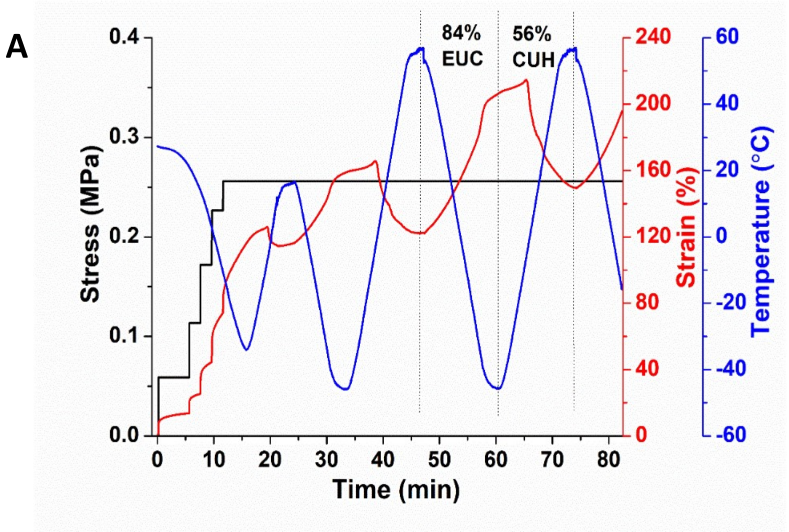

B

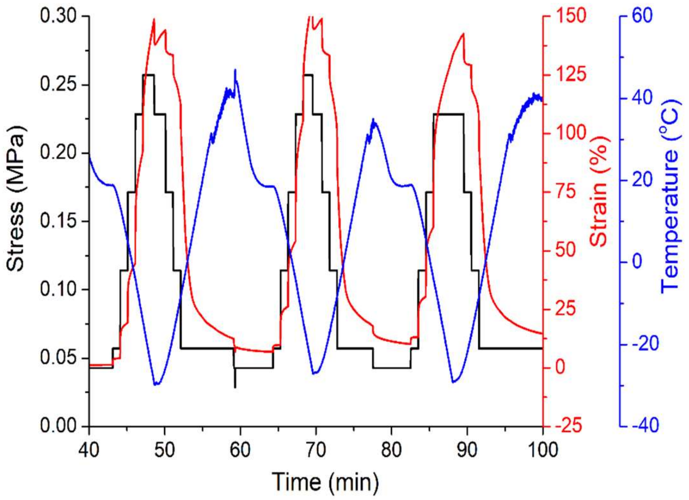

Figure S18. The "real world simulation" for cured Budene 1208 after installation. (A) The 2W-SMP experienced cooling-induced tensile stress, and then demonstrated 2W-SME. (B) Simulation of daily temperature cycle, accompanied by loading and unloading. The 2W-SME and loading/unloading effect may be coupled.

16. Comparison of 2W-SMPs

The comparison of our system with the previous developed 2W-SMPs is shown in the Table S5. It is clear that our polymer shows the highest expansion (153%) with the lowest temperature (-50 °C).

Table S5. Comparison of 2W-SMPs

2W-SMP System	Load	Cooling induced elongation	Heating induced contraction	Actuation temperature window	Ref
Our system	0.18 MPa	153%	115%	-50 to 60 °C	
Crosslinked poly(Ɛ –caprolactone) (PCL)	0.45 MPa	79.1%	74.8%	-10 to 70 °C	4
Crosslinked PCL	1 MPa	22.8%	21.4%	-5 to 65 °C	5
Crosslinked PCL	0.8 MPa	40%	40%	25 to 75 °C	6
Partial crosslinked PCL	0	15%	15%	40 to 70 °C	7
polypentadecalactone- and PCL network	0.67 MPa	55%	55%	0 to 100 °C	8
Oligo(Ɛ-caprolactone) and n-butyl acrylate copolymer	0	17.1%	17.1%	0 to 37 °C	9
PCL-gelatin bilayer film	0	unfold	fold	20 to 80 °C	10
PCL-POSS double network	0.23 N	~5%	~5%	30 to 110 °C	11
Polydopamine, PCL and diisocyanate network	2 MPa	5%	5%	0 to 100 °C	12

PCL-co-poly(ethylene glycol) foam	Compress until prestrain 30%	-15%	-15%	-20 to 80 °C	13
Crosslinked oligo(pentadecalactone)	18.2 kA m^{-1}	35%	35%	Magnetic field on & off	14
Oligo(pentadecalactone) and magnetic NPs composites	17 kA m^{-1}	45%	45%	Magnetic field on & off	15
Modeling of high density polyethylene	1 MPa	60%	60%	16 to 157 °C	16
Carbon black in crosslinked polyethylene	0.855 MPa	66.6%	62.3%	45 to 130 °C	17
Nafion	0.47 MPa	~15%	~10%	80 to 120 °C	18
Poly(octylene adipate)	0	36%	36%	25 to 56 °C	19
Poly(octylene adipate)	0	fold	unfold	5 to 38 °C	20
Poly(octylene adipate)-co-poly(octylene diazoadipate)	0	taller	shorter	25 to 45 °C	21
Crosslinked polycyclooctene	700 kPa	~25%	~25%	15 to 70 °C	22-23
Crosslinked poly(cyclooctene)	0.6 MPa	120%	120%	0 to 100 °C	24
Epoxy polymer composite	0.3 N	recovery	N/A	20 to 150 °C	25
Epoxy polymer film with high Tg surround core with low Tg	N/A		Buckling	148 to 209 °C	26
SMP composite (2W-SMP in an elastomeric matrix)	0<br>0.7 MPa	10%<br>25%	10%<br>25%	15 to 70 °C	27
Crosslinked poly(ethylene-co-vinyl acetate) (PEVA)	1.125 kPa	~30%	~30%	0 to 105 °C	28
Crosslinked PEVA	0	12%	12%	25 to 75 °C	29
Laminate of a resin plate on a fiber-reinforced polymer plate	0	straighten	bend	25 to 80 °C	30
Polyurethane (PU)	0.445 MPa	~80%	~80%	20 to 65 °C	31
Polymer laminate (100% strain PU and as-prepared PU layers)	0	straighten	bend	25 to 60 °C	29,32-33
Crosslinked PCL based PU	0.4 MPa	44%	37%	0 to 65 °C	34
Poly(ester urethane)	1.25 MPa	30%	30%	-20 to 60 °C	35
Copolyester urethane of poly(pentadecalatone) and PCL	0	20%	20%	0 to 50 °C	36
Liquid crystalline polyester	36.7 kPa	70%	70%	110 to 162 °C	37

Movie S1: The dynamic process of the reversible actuation of the cPBD under cooling and heating with 4x speed.

References

1. Fan, J. & Li, G. High enthalpy storage thermoset network with giant stress and energy output in rubbery state. *Nat. Commun.* **9**, 642 (2018).
2. Chung, T., Romo-Uribe, A. & Mather, P. T. Two-way reversible shape memory in a semicrystalline network. *Macromolecules* **41**, 184-192 (2008).
3. Ma, L. *et al.* Effects of carbon black nanoparticles on two-way reversible shape memory in crosslinked polyethylene. *Polymer* **56**, 490-497 (2015).
4. Huang, M. et al. Two-way shape memory property and its structural origin of cross-linked poly(ε-caprolactone). RSC Adv. 4, 55483-55494, (2014).

5. Pandini, S. et al. Two-way reversible shape memory behavior of crosslinked poly(ε-caprolactone). Polymer 53, 1915-1924, (2012).
6. Pandini, S. et al. The two-way shape memory behaviour of crosslinked poly(ε-caprolactone) systems with largely varied network density. J. Intell. Mater. Syst. Struct., (2015).
7. Meng, Y., Jiang, J. & Anthamatten, M. Shape actuation via internal stress-induced crystallization of dual-cure networks. ACS Macro Lett. 4, 115-118, (2015).
8. Zotzmann, J., Behl, M., Hofmann, D. & Lendlein, A. Reversible triple-shape effect of polymer networks containing polypentadecalactone- and poly(ε-caprolactone)-segments. Adv. Mater. 22, 3424-3429, (2010).
9. Saatchi, M., Behl, M., Nöchel, U. & Lendlein, A. Copolymer networks from oligo(ε-caprolactone) and n-butyl acrylate enable a reversible bidirectional shape-memory effect at human body temperature. Macromol. Rapid Commun. 36, 880-884, (2015).
10. Stroganov, V. et al. Reversible thermosensitive biodegradable polymeric actuators based on confined crystallization. Nano Lett. 15, 1786-1790, (2015).
11. Lee, K. M., Knight, P. T., Chung, T. & Mather, P. T. Polycaprolactone−POSS chemical/physical double networks. Macromolecules 41, 4730-4738, (2008).
12. Bai, Y., Zhang, X., Wang, Q. & Wang, T. A tough shape memory polymer with triple-shape memory and two-way shape memory properties. J. Mater. Chem. A 2, 4771-4778, (2014).
13. Baker, R. M., Henderson, J. H. & Mather, P. T. Shape memory poly(?-caprolactone)-co-poly(ethylene glycol) foams with body temperature triggering and two-way actuation. J. Mater. Chem. B 1, 4916-4920, (2013).
14. Razzaq, M. Y., Behl, M., Kratz, K. & Lendlein, A. Multifunctional hybrid nanocomposites with magnetically controlled reversible shape-memory effect. Adv. Mater. 25, 5730-+, (2013).
15. Razzaq, M. Y., Behl, M., Nöchel, U. & Lendlein, A. Magnetically controlled shape-memory effects of hybrid nanocomposites from oligo(ω-pentadecalactone) and covalently integrated magnetite nanoparticles. Polymer 55, 5953-5960, (2014).
16. Dolynchuk, O., Kolesov, I. & Radusch, H.-J. Thermodynamic description and modeling of two-way shape-memory effect in crosslinked semicrystalline polymers. Polym Adv. Technol. 25, 1307-1314, (2014).
17. Ma, L. et al. Effects of carbon black nanoparticles on two-way reversible shape memory in crosslinked polyethylene. Polymer 56, 490-497, (2015).
18. Xie, T., Li, J. & Zhao, Q. Hidden thermoreversible actuation behavior of Nafion and its morphological origin. Macromolecules 47, 1085-1089, (2014).
19. Tippets, C. A. et al. Dynamic optical gratings accessed by reversible shape memory. ACS Appl. Mater. Interfaces 7, 14288-14293, (2015).
20. Zhou, J. et al. Shapeshifting: Reversible shape memory in semicrystalline elastomers. Macromolecules 47, 1768-1776, (2014).
21. Turner, S. A., Zhou, J., Sheiko, S. S. & Ashby, V. S. Switchable micropatterned surface topographies mediated by reversible shape memory. ACS Appl. Mater. Interfaces 6, 8017-8021, (2014).
22. Westbrook, K. K. et al. Constitutive Modeling of Shape Memory Effects in Semicrystalline Polymers With Stretch Induced Crystallization. Journal of Engineering Materials and Technology 132, 041010-041010, (2010).
23. Qi, G., Kristofer, K. W., Patrick, T. M., Martin, L. D. & Qi, H. J. Thermomechanical behavior of a two-way shape memory composite actuator. Smart Mater. Struct. 22, 055009, (2013).
24. Chung, T., Romo-Uribe, A. & Mather, P. T. Two-way reversible shape memory in a semicrystalline network. Macromolecules 41, 184-192, (2008).

25. Basit, A., Hostis, G., Pac, M. & Durand, B. Thermally activated composite with two-way and multi-shape memory effects. Materials 6, 4031, (2013).
26. Wang, Z., Song, W., Ke, L. & Wang, Y. Shape memory polymer composite structures with two-way shape memory effects. Mater. Lett. 89, 216-218, (2012).
27. Kristofer, K. W. et al. Two-way reversible shape memory effects in a free-standing polymer composite. Smart Mater. Struct. 20, 065010, (2011).
28. Li, J., Rodgers, W. R. & Xie, T. Semi-crystalline two-way shape memory elastomer. Polymer 52, 5320-5325, (2011).
29. Behl, M., Kratz, K., Noechel, U., Sauter, T. & Lendlein, A. Temperature-memory polymer actuators. P. Natl. Acad Sci. 110, 12555-12559, (2013).
30. Tamagawa, H. Thermo-responsive two-way shape changeable polymeric laminate. Mater. Lett. 64, 749-751, (2010).
31. Seok Jin, H., Woong-Ryeol, Y. & Ji Ho, Y. Two-way shape memory behavior of shape memory polyurethanes with a bias load. Smart Mater. Struct. 19, 035022, (2010).
32. Chen, S., Hu, J., Zhuo, H. & Zhu, Y. Two-way shape memory effect in polymer laminates. Mater. Lett. 62, 4088-4090, (2008).
33. Chen, S., Hu, J. & Zhuo, H. Properties and mechanism of two-way shape memory polyurethane composites. Compos. Sci. Technol. 70, 1437-1443, (2010).
34. Raquez, J.-M. et al. Design of cross-linked semicrystalline poly(ε-caprolactone)-based networks with one-way and two-way shape-memory properties through diels–alder reactions. Chem. Eur. J. 17, 10135-10143, (2011).
35. Bothe, M. & Pretsch, T. Two-way shape changes of a shape-memory poly(ester urethane). Macromol. Chem. Phys. 213, 2378-2385, (2012).
36. Behl, M., Kratz, K., Zotzmann, J., Nöchel, U. & Lendlein, A. Reversible bidirectional shape-memory polymers. Adv. Mater. 25, 4466-4469, (2013).
37. Qin, H. & Mather, P. T. Combined one-way and two-way shape memory in a glass-forming nematic network. Macromolecules 42, 273-280, (2009).
